# Quantifying Small-Molecule Association with Lipid Membranes: Methods, Models, and Limitations

**DOI:** 10.3390/membranes16070218

**Published:** 2026-06-26

**Authors:** Maria João Moreno, Margarida M. Cordeiro, Hugo A. L. Filipe, Alexandre C. Oliveira, Cristiana L. Pires, Cristiana V. Ramos, Jaime Samelo, Jorge Martins, Luís M. S. Loura

**Affiliations:** 1 Coimbra Chemistry Center, Institute of Molecular Sciences (CQC-IMS), University of Coimbra, 3004-535 Coimbra, Portugal; mmc.margarida0@gmail.com (M.M.C.); hlfilipe@ipg.pt (H.A.L.F.); ac_oliveira10@hotmail.com (A.C.O.); cristiana.lages.pires@gmail.com (C.L.P.); cristianavramos95@gmail.com (C.V.R.); jaimesamelo@gmail.com (J.S.); lloura@ff.uc.pt (L.M.S.L.); 2 Department of Chemistry, Faculty of Sciences and Technology, University of Coimbra, 3004-535 Coimbra, Portugal; 3 BRIDGES—Biotechnology Research, Innovation, and Design of Health Products, Polytechnic University of Guarda, 6300-559 Guarda, Portugal; 4 Centro de Ciências do Mar (CCMAR/CIMAR, LA) and DCBB-FCT, Universidade do Algarve, 8005-139 Faro, Portugal; jmartin@ualg.pt; 5 Faculty of Pharmacy, University of Coimbra, 3000-548 Coimbra, Portugal

**Keywords:** biomembranes, partition coefficient, drug–membrane association, drug design

## Abstract

The association of small molecules with lipid membranes plays a central role in drug delivery, pharmacokinetics, toxicity, and membrane biophysics, also being of fundamental importance in drug pharmacodynamics given that most drug targets are membrane-associated proteins. Accurate determination of solute–membrane association affinities, however, remains challenging due to the diversity of experimental systems, the complexity of membrane environments, and the intrinsic limitations of individual methodologies. This review provides a comprehensive overview of the experimental and computational approaches currently used to quantify small molecule association with lipid membranes. Standard experimental techniques, including spectroscopy-based methods, calorimetry, electrophoretic measurements, and surface-sensitive approaches, are discussed alongside established computational strategies ranging from continuum models to atomistic molecular dynamics simulations. Particular emphasis is placed on the formalisms required for data analysis, including partitioning models and thermodynamic frameworks, as well as on the assumptions underlying each method. The validity limits, sources of uncertainty, and common experimental and interpretative pitfalls are critically examined. By providing a unified and comparative perspective, this work establishes a structured framework for the quantitative study of solute–membrane interactions, guiding new researchers in the selection of appropriate methodologies and in the rigorous analysis of experimental and computational results. Moreover, it enables the consistent and quantitative rationalization of affinity parameters reported across the literature, supporting the development of curated datasets and predictive relationships that can inform the design of new and more effective drugs.

## 1. Introduction

The association of drugs with biological membranes is one of the key steps that determines drug bioavailability and pharmacokinetics. The importance of this property is well recognized and is usually estimated from the drug’s partition between water and octanol due to its simpler experimental evaluation. However, octanol is unable to capture the distinctive features of biological membranes. On one hand, the polarity of octanol is much higher than that of the nonpolar bilayer center and much lower than that of the polar headgroups, lacking the charged groups present in phospholipids, the most abundant lipids in biomembranes. Octanol is therefore unable to mimic the membrane interaction of ionized drugs or any process that is influenced by membrane electrostatic potentials [1,2]. Moreover, while octanol polarity is comparable to the average polarity of lipid bilayers and similar to the region of intermediate polarity where the energy minimum is located for most drugs [3,4,5,6,7,8,9,10,11], the H bonding ability is different. Together with the absence of charges, this leads to different relative stabilities of the protonated/unprotonated species of drugs with ionizable groups, which strongly influences their partition and permeability.

Other water-immiscible organic solvents have been used to mimic the membrane affinity of small uncharged solutes [12]. However, no organic solvent or their combinations may capture the solvent properties of lipid bilayers due to the distinct structural features. The membrane’s lipid bilayer is a structured, very thin, and strongly anisotropic media, changing from a highly polar and charged headgroup region in contact with water to the nonpolar center of the bilayer in just a few nanometers [13,14]. Because the thickness of each monolayer is comparable to the dimensions of most bioactive molecules, they span membrane regions of very distinct solvation properties, adopting conformations, locations, and orientations that maximize favorable interactions with the membrane [5,8,10,15,16,17,18,19,20,21,22,23]. It has been well recognized for many decades (e.g., [24,25]) that lipid bilayers are interfacial phases, and the membrane partition is of a fundamentally different nature than the partition into bulk phases. In this context, it is worth noting that octanol is not truly a homogeneous medium at the nanoscopic scale, being organized as inverted micelles with H-bonds between the hydroxyl groups stabilizing the micelle core [26,27,28]. The size of the micelle polar core increases in water-saturated octanol, which, at room temperature, contains over 2 M water [29,30]. The high water content and local structure increase the solubility of polar molecules [30] and are likely the reasons for its moderate success in predicting associations with proteins and lipid membranes [31], although the lack of charged groups and distinct H-bonding ability limits its mimicking capacity.

The inadequacy of nonpolar solvents as models of biomembranes is one of the reasons for the poor predictivity of drugs’ pharmacokinetics, namely their ability to cross tight endothelia such as the blood–brain barrier [32,33,34,35,36,37,38,39]. Membrane partition is also a major determinant in the pharmacodynamics of many drugs, with their membrane affinity as well as their location and orientation in the membrane strongly influencing their interaction with membrane proteins.

Over the last century, a large number of studies have been performed regarding drug–membrane interactions. It is of the highest importance to take advantage of these data to improve the predictive ability of membrane affinity from a drug’s structure and molecular properties. What is delaying this important task is the lack of uniformization of the formalisms used to quantitatively describe the drug/membrane interaction [40,41,42], and their organization in a publicly available database containing the relevant information, manually curated, reliable, and ready to use.

There are several publications where some specific methodologies to study drug/membrane association have been reviewed. Among them, it is worth noting the work by Santos et al. [42], which gives an extensive description of the optical spectroscopic methods, with important information regarding experimental details and possible artifacts. Other important reviews can also be found for the application of optical spectroscopic methodologies [43,44,45], calorimetry [46,47], potentiometry, dialysis, and NMR [48] and for the use of molecular dynamics simulations [49,50]. Each specific approach has its own particularities, namely regarding the membrane properties and the concentration of drug, but also the parameters to quantify the drug–membrane affinity. To allow the use of the information collectively obtained by the scientific community, it is necessary to establish consensus parameters and conditions, as well as quantitative relationships to convert the information directly obtained into the consensus parameters. This will maximize the information available and allow its use for artificial intelligence applications to find rules and quantitative relationships to predict the membrane affinity from the drug’s molecular properties.

The objective of this review is to combine the information from all available methodologies to characterize the association of small molecules with lipid membranes. Each methodology is documented with some applications, and its strengths and weaknesses are summarized. We first discuss the formalisms used to quantify the membrane affinity, establishing the quantitative relations between them and their application limits. This allows the comparison between results obtained with different approaches and guides new researchers in the field in their selection for the most appropriate approach. Key references are provided for the distinct methodologies and formalisms. The list of references, however, is not exhaustive, and the authors apologize in advance for the omission of many important works. This review will set the framework for the implementation of all available data on the interaction of small molecules with lipid membranes in its future inclusion in a publicly available database.

## 2. Parameters to Quantify the Affinity for the Membrane

Several parameters can be used to quantify the affinity of a small molecule for lipid membranes. To facilitate comparison between this measure of molecular lipophilicity and the commonly used water/octanol partition coefficient, the reference parameter adopted in this review is the ratio of the solute molar concentrations in the two phases (aqueous and membrane), as defined in Equation (1):

(1) $$\begin{array}{c} n_{S_{W}}\begin{array}{c} \overset{K_{P}V_{M}}{↔} \end{array}\;n_{S_{M}}\\ K_{P}=\frac{n_{S_{M}}/V_{M}}{n_{S_{W}}/V_{W}} \end{array}$$

In this equation, $n_{S_{M}}\;$is the moles of solute in the membrane phase, which has a total volume $V_{M}$, and $n_{S_{W}}\;$is the moles of solute in the aqueous phase with the total volume $V_{W}$.

Other approaches have been considered in the literature for the quantification of the affinity of the solute for the membrane. The most common include the partition in terms of solute concentration expressed in molality ($K_{P}^{n/m}$, Equation (2)), molar fraction ($K_{P}^{n/n}$, Equation (3)), or mass ratio ($K_{P}^{m/m}$, Equation (4)) of solute in each phase. The distinct parameters are usually reported as partition coefficients without explicit indication in the parameter name of the formalism followed. Care must therefore be taken in the identification of the parameter being reported through the analysis of the equation used for its estimation. This is especially important given that all these different approaches lead to dimensionless affinity parameters, requiring careful reading of the original report for correct identification.

(2) $$K_{P}^{n/m}=\frac{n_{S_{M}}/\left(m_{L}+m_{S_{M}}\right)}{n_{S_{W}}/\left(m_{W}+m_{S_{W}}\right)}$$

(3) $$K_{P}^{n/n}=\frac{n_{S_{M}}/\left(n_{L}+n_{S_{M}}\right)}{n_{S_{W}}/\left(n_{W}+n_{S_{W}}\right)}$$

(4) $$K_{P}^{m/m}=\frac{m_{S_{M}}/\left(m_{L_{M}}+m_{S_{M}}\right)}{m_{S_{W}}/\left(m_{W}+m_{S_{W}}\right)}$$

where $m_{L}$, $m_{W}$, $m_{S_{M}}$ and $m_{S_{W}}$ are the mass of lipid in the membrane phase, water in the aqueous phase, solute in the membrane phase, and solute in the aqueous phase, respectively, and $n_{L}$ and $n_{W}$ are the number of moles of lipid in the membrane and the number of moles of water in the aqueous phase, respectively.

If both the aqueous and membrane phases are below saturation with solute, the different parameters are interconvertible. In this case, the solute concentration is usually much lower than that of the lipid, and the total volume (or mass) of the membrane phase may be represented by that from the lipids only. The different parameters are in this case related by the following:

(5) $$K_{P}=K_{P}^{n/m}\frac{\rho_{M}}{\rho_{W}}=K_{P}^{n/n}\frac{1}{\bar{V_{L}}\left[W\right]}$$

where $\rho_{M}$ and $\rho_{W}$ are the densities of the membrane and aqueous phase, respectively. The transversal density of the lipid bilayer is very heterogeneous, being higher in the region of the polar head group and smaller in the midplane of both membrane leaflets [51,52]. The overall density is however close to 1 g/cm^3^ [51,52,53,54], and therefore$\;K_{P}≅K_{P}^{n/m}$. For a given affinity parameter, the fraction of solute associated with the membranes (in conditions below saturation of both the aqueous phase and the membrane) may be calculated using Equation (6):

(6) $$\frac{n_{S_{M}}}{n_{T}}=\frac{K_{P}\bar{V_{L}}{\left[L\right]}_{\mathrm{Molar}}}{1+K_{P}\bar{V_{L}}{\left[L\right]}_{\mathrm{Molar}}}=\frac{K_{P}^{n/m}{\left[L\right]}_{\mathrm{molal}}}{1+K_{P}^{n/m}{\left[L\right]}_{\mathrm{molal}}}=\frac{K_{P}^{n/n}{\left[L\right]}_{\mathrm{Molar}}}{\left[W\right]+K_{P}^{n/n}{\left[L\right]}_{\mathrm{Molar}}}$$

where ${\left[L\right]}_{x}$ is the concentration of lipid relative to the total solution (the subscript x denoting the concentration units), $\left[W\right]\;$is the concentration of water in the aqueous phase (approximately 55.5 M), and $\bar{V_{L}}$ is the molar volume of the lipids in the membrane. The latter is the key property that allows the conversion of the affinity parameters into the partition coefficient$\;K_{P}$. This property depends on the lipid composition, namely on the lipid molar mass and the membrane phase. For a certain lipid organized as a lipid bilayer, the molar volume is larger when the membrane is in the liquid–crystalline phase and smaller when in the gel or liquid-ordered phases. The specific volume of phosphatidylcholines in the gel phase is around 0.95 mL/g, increasing to around 1.0 mL/g in the fluid phase [55,56], corresponding to a molar volume of 0.70 and 0.74 dm^3^/mol for DPPC in the gel and fluid phases, respectively. Increasing the length of the acyl chains adds 27 Å^3^ per methylene group [57], which corresponds to a rise of 0.02 dm^3^/mol, while the introduction of cis double bonds results in only a slight increase in the molar volume (smaller than 0.01 dm^3^/mol per double bond [56]). The most abundant acyl chains in the phospholipids present in biomembranes contain 16 to 20 carbons [58,59,60,61,62], leading to an average value of $\bar{V_{L}}$ close to 0.8 dm^3^/mol. If the membranes contain high amounts of sterols, the molar volume is significantly lower due to the smaller size of the sterols and to the condensing effect induced by cholesterol in the lipid acyl chains. As observed for the effect of phase transition on the lipid molar volume, the effect of cholesterol condensation itself is relatively small (a decrease of ca. 2% for saturated acyl chains and even smaller for lipids with *cis* double bonds [56]). Therefore, the effects on the average lipid molar volumes are mostly due to changes in the volumes of each lipid and may be considered additive. The molar volume estimated for cholesterol in a lipid membrane is 0.38 dm^3^/mol [63]. At 20 mol% cholesterol in the membrane, the average lipid molar volume is therefore decreased by 10%, and at cholesterol-saturating concentrations (ca. 50 mol% [64,65]) it is decreased by 26%, leading to $\bar{V_{L}}$ = 0.6 dm^3^/mol. For simplicity, in the conversion of partition coefficients from molar fraction ($K_{P}^{n/n}$) into the consensus partition coefficient ($K_{P}$), $\bar{V_{L}}$ = 0.8 dm^3^/mol is suggested for lipid membranes in the gel or in the liquid disordered phase (less than 20 mol% cholesterol) and $\bar{V_{L}}$ = 0.6 dm^3^/mol for lipid membranes in the liquid ordered phase (more than 30 mol% cholesterol).

The treatment of the lipid membrane as a phase immiscible with water leads to the above formalisms, where the association of the solute with the lipid membranes is treated as a partition coefficient. This is generally valid for the case of small molecules at low concentrations. However, for some solutes and methodologies, the association is best described by a formalism considering solute binding to the membranes instead of partition between the two phases. This is particularly relevant when the solutes are large and present several charged groups, as is the case of polycationic peptides and proteins [66]. In this situation, it may be relevant to consider the presence of binding sites in the membrane, as well as the decrease in the binding capacity due to solute binding. The kinetic scheme and the equilibrium binding constant for solute binding to independent binding sites in the membrane may be defined by Equation (7):

(7) $$\begin{array}{c} S_{W}+B\;\begin{array}{c} \overset{K_{b}}{↔} \end{array}\;S_{M}\\ K_{b}=\frac{\left[S_{M}\right]}{\left[S_{W}\right]\left[B\right]}\;;\;\left[S_{W}\right]+\left[S_{M}\right]=\left[S_{T}\right]\;;\;\left[B\right]=\frac{\left[L_{T}\right]}{{\#}_{L}}-\left[S_{M}\right] \end{array}$$

where $K_{b}$ is the binding constant, $\left[S_{W}\right]\;$and $\left[S_{M}\right]$ correspond to the solute concentration free in the aqueous phase and associated with the lipid membrane, respectively, $\left[B\right]$ is the concentration of free binding sites (all with respect to the total volume of the solution), $\left[L_{T}\right]$ is the total concentration of lipid, and ${\#}_{L}$ is the number of lipid molecules per binding site. Equation (7) lead to a quadratic equation, where the physically meaningful solution is always $x_{-}$ (Equation (8)).

(8) $$\left[S_{M}\right]=\frac{-b-\sqrt{b^{2}-4ac}}{2a};a=K_{b};\;b=-1-K_{b}\left(\frac{\left[L_{T}\right]}{{\#}_{L}}+\left[S_{T}\right]\right);c=K_{b}\left[S_{T}\right]\frac{\left[L_{T}\right]}{{\#}_{L}}$$

In the case of low affinity binding where only a negligible fraction of solute is associated with the membrane and when the affinity is evaluated from the dependence of $S_{M}$ with $S_{T}$, $K_{b}$ may be estimated from the simplified Equation (9):

(9) $$K_{b}≅\frac{\left[S_{M}\right]}{\left[S_{T}\right]\left(\frac{\left[L_{T}\right]}{{\#}_{L}}\;-\;\left[S_{M}\right]\right)};\;\left[S_{M}\right]≅\frac{K_{b}\left[S_{T}\right]\frac{\left[L_{T}\right]}{{\#}_{L}}}{1+K_{b}\left[S_{T}\right]}$$

The binding constant is related to the partition coefficient by Equation (10) [67].

(10) $$K_{P}=\frac{K_{b}}{{\#}_{L}\bar{V_{L}}}\;$$

In most publications following this approach, the number of lipids per binding site is not explicitly assumed. This is equivalent to the assumption of ${\#}_{L}$ = 1, and the resulting binding constant is usually referred to as $K_{L}$.

Another association parameter that has been used to quantify the affinity of solutes for lipid membranes results from considering the whole lipid vesicle as the binding agent. This formalism is used when the equilibrium affinity is obtained from the ratio of the association and dissociation rate constants, calculated from the dependence of the rate of association with the concentration of lipid vesicles, and is relevant to evaluate if solute–membrane association is a diffusion-limited process [68,69,70,71,72]. In this case, the equilibrium constant is given by Equation (11) and related with the corresponding partition coefficient by Equation (12).

(11) $$\begin{array}{c} S_{W^{o}}+L_{V}\;\begin{array}{c} k_{+}^{L_{V}}[L_{V}]\\ ⇌\\ k_{-} \end{array}{\;S}_{L^{o}}\\ K_{L_{V}}=\frac{k_{+}^{L_{V}}}{k_{-}}=\frac{\left[S_{L^{o}}\right]}{\left[S_{W^{o}}\right]\left[L_{V}\right]}\;;\;\left[L_{V}\right]=\frac{\left[L_{T}\right]}{n_{L/L_{V}}} \end{array}$$

(12) $$K_{P}=\frac{K_{L_{V}}}{{\bar{V}}_{L_{V}^{o}}}=\frac{K_{L_{V}}}{n_{L/L_{V}^{o}}{\bar{V}}_{L}}$$

where $S_{W^{o}}$ is the solute in the aqueous media outside the vesicles, $S_{L^{o}}$ is the solute associated with the outer leaflet of the vesicle membrane, $\left[L_{V}\right]$ is the concentration of the lipid vesicles, ${\bar{V}}_{L_{V}^{o}}$ is the volume of lipid in the outer leaflet of one mole of vesicles, which is equal to the product of the number of lipid molecules in the leaflet ($n_{L/L_{V}^{o}}$) and the lipid molar volume. The latter is calculated from the size of the vesicles and the area per lipid, which in the fluid phase is 60 to 70 Å^2^ for phosphatidylcholine, sphingomyelin, and phosphatidylserine [73,74,75] and 55 to 65 Å^2^ for phosphatidylethanolamine [76,77], the area increasing with the number of cis unsaturations in the acyl chains [74,78], and being smaller when the membrane is in the gel or liquid ordered phases [74,76,78,79]. A cross-sectional area of 39 Å^2^ is usually considered for cholesterol, independently of the membrane phase [80].

In early work, there was another additional measure used to quantify the affinity of the solute to the membrane, which was defined as the thickness of the aqueous medium in contact with the surface of the membrane that contained the same moles of solute as found associated with the membrane [81]. This affinity parameter has units of length and is related to the partition coefficient by Equation (13):

(13) $$K=K_{P}\;l$$

where l is the thickness of the membrane to which the solute equilibrates (the whole bilayer if the solute equilibration between the two membrane leaflets is fast). Typical values for the thickness of fully hydrated bilayers composed of phospholipids with 16 carbons in the acyl chains are close to 5 nm, being somewhat larger in the gel than in the fluid phase [76]. Shorter/longer acyl chains lead to bilayers with lower/higher thickness [82]. The exact value reported for the bilayer thickness depends on the lipid groups considered, methodology used, and lipid composition [51,57,73,83,84,85,86,87,88]. Nevertheless, 5 nm can usually be considered as a reasonable estimate for bilayers prepared from the phospholipids usually found in biomembranes. The use of *K* as a quantitative parameter for the drug–membrane affinity may be of particular interest when evaluating the effect of lipophilicity on permeability and using membrane systems with large unstirred water layers (e.g., permeability assays using cell monolayers [89,90,91,92,93,94]).

### 2.1. Effect of Electrostatic Interactions and Ionic Strength on the Observed Solute–Membrane Affinity

In the partition formalisms described above, it is assumed that the solute does not affect the membrane properties, and therefore the solute affinity for the membrane is not dependent on its concentration. In this case, it is not necessary to include changes in the membrane volume (or mass) nor in the membrane’s charge or solvation properties. This is not the case at high solute concentrations, especially when the solute is a lipid-like molecule that becomes a constituent of the membrane and may achieve very high local concentrations [47]. Due to the changes in the membrane’s properties, the partition coefficient obtained will depend on solute concentration. For the comparison of different solutes and rationalization of the relation between the affinity for the membrane and solute structural properties, it is necessary to consider the affinity for an unperturbed membrane. In the case of charged solutes, it may also be relevant to distinguish between the contribution of electrostatic interactions (repulsive or attractive interactions with the charged lipid headgroups or solute-solute repulsive interactions) and hydrophobic interactions established with the nonpolar portion of the membrane. Although both types of interactions contribute to the affinity observed, they depend differently on the solute concentration and on system properties such as the lipid type, pH or ionic strength. By knowing both contributions separately, well-established formalisms such as the Gouy–Chapman theory may be used to calculate the partition coefficient at conditions other than those evaluated experimentally.

The observed partition coefficient ($K_{P}$) is related to the partition coefficient in the absence of electrostatic effects ($K_{P}^{0}$), by the membrane surface potential ($\psi_{0}$) and the solute charge ($z_{S}$), Equation (14):

(14) $$K_{P}=K_{P}^{0}\;e^{-\frac{z_{S}F\psi_{0}}{RT}}\equiv \;K_{P}^{0}=K_{P}\;e^{\frac{z_{S}F\psi_{0}}{RT}}$$

where F is the Faraday constant (96485 C/mol), R is the molar gas constant (8.314 J K^−1^ mol^−1^), and T is temperature in Kelvin. This dependence of the observed affinity on the electrostatic interaction of the solute with the membrane is due to the accumulation (or depletion) of solute at the water–membrane interface, the aqueous media in the immediate vicinity of the membrane from which the solute partitions into the membrane, ${\left[S_{W}\right]}_{\mathrm{inter}}$ [95], from Equation (15):

(15) $${\left[S_{W}\right]}_{\mathrm{inter}}={\left[S_{W}\right]}_{\mathrm{bulk}}e^{-\frac{z_{S}F\psi_{0}}{RT}}$$

After calculation of $K_{P}^{0}$ for the specific experimental conditions used to characterize the solute affinity, the observed partition coefficient ($K_{P}$) may be calculated at any other condition provided that the electrostatic interaction is the only different variable (namely a different solute concentration while at non-perturbing conditions, a different density of charged lipids in the membrane, or at a distinct ionic strength in the aqueous media).

To calculate $K_{P}^{0}$ from $K_{P}$ using Equation (14), it is necessary to know the membrane surface potential. This property is related to the density of charged species in the membrane ($\sigma_{0}$) by Equation (16) [95,96,97]:

(16) $$\sigma_{0}=\frac{\psi_{0}}{\left|\psi_{0}\right|}\sqrt{2000\epsilon_{0}\epsilon_{r}RT\sum_{j}C_{j}\left(e^{-\frac{z_{j}F\psi_{0}}{RT}}-1\right)}$$

where $\epsilon_{0}$ is the vacuum permittivity (8.85 × 10^−12^ F/m) and $\epsilon_{r}$ is the relative permittivity of the medium where the surface potential is calculated (usually considered to be equal to that of the aqueous media, 78 at 25 °C). The sum in Equation (16) is extended to all ions present in the aqueous media, with a concentration $C_{j}$ and charge $z_{j}$.

The membrane charge density may also be calculated directly from the charges present at the membrane surface:

(17) $$\sigma_{0}=e_{0}\frac{\sum_{i}z_{i}n_{i}}{\sum_{i}A_{i}n_{i}}$$

where $e_{0}$ is elementary electrostatic charge (1.60 × 10^−19^ C), the sum $\sum_{i}z_{i}n_{i}$ extends to all charges in the membrane (lipids, solute and eventual ions adsorbed), and $\sum_{i}A_{i}n_{i}$ is the membrane surface area (including only the lipids if the local solute concentration is low, or both lipids and solutes if the solute molar fraction is significant [3,98]). The amount of solute in the membrane is in turn calculated from Equation (6) and the value of $K_{P}$ calculated from Equation (14), providing an independent link between $\sigma_{0}$ and $\psi_{0}$. The charge density at the membrane surface depends on the affinity of the solute towards the membrane, and, in turn, the solute affinity depends on the membrane surface charge density. The membrane affinity of the solute must therefore be obtained from the convergence of the values of $\sigma_{0}$ calculated from Equations (16) and (17) at different values of $\psi_{0}$ and $K_{P}^{0}$ (and corresponding $K_{P}$).

For small surface potentials ($\left|\psi_{0}\right|$ < 25 mV) and electrolyte solutions mostly composed by monovalent ions, Equation (16) may be simplified to Equation (18) [99,100], where $\lambda_{D}$ is the Debye length, and I is the ionic strength.

(18) $$\psi_{0}=\sigma_{0}\frac{\lambda_{D}}{\epsilon_{0}\epsilon_{r}};\lambda_{D}=\sqrt{\frac{RT\epsilon_{0}\epsilon_{r}}{2F^{2}I}}$$

This allows the direct calculation of the surface potential from the surface charge density obtained by Equation (17), without the need for iterative procedures until convergence [3].

When collecting partition coefficients from the literature with the goal of establishing rules between the solute–membrane affinity and solute structural properties, both $K_{P}^{0}$ and $K_{P}$ are relevant. The former is independent on the solute and membrane charges (allowing the evaluation of the effects of solute properties other than its charge), while the latter reflects the effective affinity for some specific conditions (membrane charge and solute ionization and concentration).

**Figure 1:**
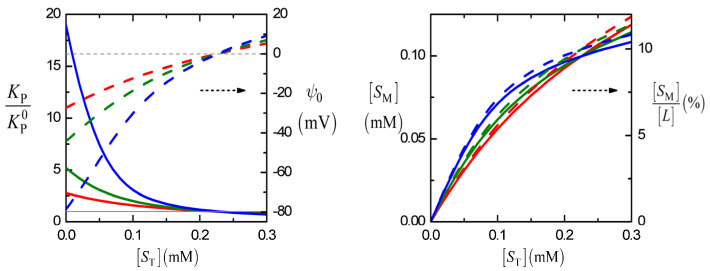
Effect of the concentration of a solute with $z_{S}$ = +1 on the observed affinity ($K_{P}$) for membranes containing 10 mol% of negatively charged lipids, an ionic strength of 0.15 M (red), 0.05 M (green), or 0.01 M (blue), a moderate solute–membrane affinity $K_{P}^{0}$ = 10^3^, and a lipid concentration of 1 mM. The left plot shows the variation of the ratio between the observed affinity and the affinity for an uncharged membrane ($K_{P}/K_{P}^{0}$, continuous lines) and the variation of the membrane surface potential ($\psi_{0}$, dashed lines). The grey lines in the left plot correspond to the case of an uncharged membrane and solute ($\psi_{0}$ = 0 mV, dashed line), where $K_{P}=K_{P}^{0}$ (continuous line) at all solute concentrations. The plot on the right shows the variation of the concentration of solute associated with the membrane ($\left[S_{M}\right]$, continuous lines) and the mol% of solute relative to lipids in the membrane ($\left[S_{M}\right]/\left[L\right]$, dashed lines).

Correction for electrostatic effects is usually necessary when the membrane affinity of charged molecules is characterized in vitro due to the relatively high local concentrations of solute in the membrane achieved. To evaluate the necessity to correct for the effects of electrostatics and solute concentration when considering drug distribution between membrane compartments in vivo, one should consider the expected in vivo conditions. The amount of membranes in the blood or in the tissues is quite high, with the concentration of phospholipids being in the mM range and up to 40 mM in the brain [39,101,102,103]. On the other hand, the total concentration of a given drug is low, being typically a few tens of mg per dose, which leads to an overall concentration in the µM range (considering an average molar mass of 200 g/mol for the solute, 70 kg for the whole system weight, and a density of 1 g/mL). Therefore, at equilibrium, the local concentration of a specific drug in biomembranes is always very low, although moderate local concentrations may be attained transiently due to slow equilibration kinetics [104,105]. Another important parameter that should be considered when evaluating the effects of charged solutes on their membrane affinity is the physiological ionic strength. This parameter depends on the specific concentration of ions in the aqueous compartments but is always high, 0.15 M being a reference value. In Figure 1, the expected effect of solute concentration on the apparent affinity of a charged solute to a lipid membrane is presented. The lipid concentration considered was 1 mM, of which 10% are negatively charged lipids. The solute was considered to have a global charge of +1, and a moderate affinity for an uncharged membrane ($K_{P}^{0}$ = 10^3^).

For very low ionic strength, the membrane surface potential in the absence of solute is large (79 mV at I = 0.01 M), and it approaches zero as the ionic strength increases (being −27 mV at I = 0.15 M). The electrostatic attraction between the positively charged solute and the negatively charged membrane leads to an increase in the observed affinity ($K_{P}>K_{P}^{0}$). However, as the solute concentration increases, the charges in the membrane are neutralized and the surface potential tends to zero. This leads to a decrease in $K_{P}$ that approaches $K_{P}^{0}$. At very high solute concentrations, the fraction of charged solute in the membrane becomes higher than that of the charged lipid, leading to a positive surface potential. At these conditions, $K_{P}$ becomes lower than $K_{P}^{0}$ due to the electrostatic repulsion between the solute and the charged solute-containing membrane.

For the high ionic strength characteristic in biological fluids and solutes with a single charge, the observed solute affinity is not significantly affected by the solute concentration and is similar to that observed for an uncharged membrane (within a factor of 2, red curves). In these simulations, it is assumed that the solute does not influence the membrane properties other than its overall surface charge density and corresponding surface potential. This assumption is reasonable given the moderate local solute concentrations achieved (≲10% relative to the lipid). However, due to the low sensitivity of some methodologies, high solute concentrations may be required to characterize its membrane affinity leading to changes in the membrane properties. It is therefore important to estimate $K_{P}^{0}$ from the observed $K_{P}$ obtained experimentally, and the former should be the reference parameter from which the affinity at the relevant specific conditions may be calculated.

### 2.2. Effect of the Interaction Kinetics on the Apparent Solute–Membrane Affinity

In the formalisms presented in the previous sections, the membrane volume (and the corresponding lipid concentration) refers to the membrane portion accessible to the solute during the solute–membrane equilibration time. The equilibration of the solute between the aqueous phase and the membrane involves several steps that will limit the extent of the association during a certain time window. The effect of the kinetics of the distinct steps on the observed amount of solute associated with the membrane is addressed in this section.

**Figure 2:**
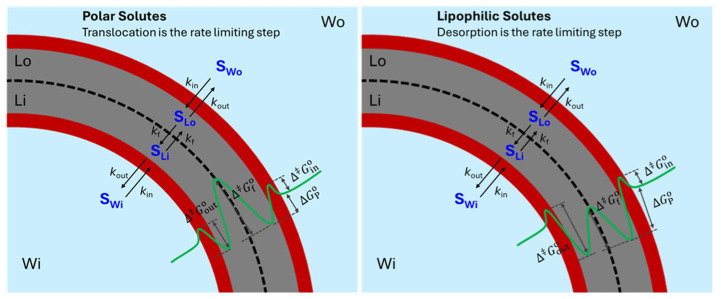
Steps in the equilibration of solute between the aqueous phase and the membrane, in the case of polar solutes where translocation is the rate-limiting step (**left plot**) and for very lipophilic solutes where desorption is the rate-limiting step (**right plot**). Variables and parameters defined in the text.

The distinct steps in the equilibration of the solute between the aqueous phase and the membrane are schematically represented in Figure 2 for the case of unilamellar liposomes. The solute may be dissolved in the aqueous media outside and inside the liposomes ($S_{Wo}$ or $S_{Wi}$, respectively) or may be associated with the outer or inner leaflet of the lipid bilayer ($S_{Lo}$ or $S_{Li}$, respectively). Depending on the solute size and polarity, the rate-limiting step may be insertion in the membrane ($k_{in}$), desorption from the membrane ($k_{out}$), or translocation (flip-flop) between the membrane leaflets ($k_{f}$). Hypothetical free energy profiles are represented by the green line, with ${\Delta G}_{P}^{o}$ indicating the free energy variation of the solute between the aqueous phase and its equilibrium position in the membrane and $\Delta^{‡}G_{X}^{o}$ the free energy variation between the distinct energy local minimum and the high energy transition states associated with the solute’s insertion $\Delta^{‡}G_{in}^{o}$, translocation $\Delta^{‡}G_{f}^{o}$, and desorption $\Delta^{‡}G_{out}^{o}$ steps.

In the case of relatively polar solutes, dissolution in the nonpolar center of the bilayer is the less favorable state, corresponding to an absolute energy maximum at this location (left plot), and translocation between the two membrane leaflets is the rate-limiting step with an energy barrier $\Delta^{‡}G_{f}^{o}$. On the other hand, for very lipophilic solutes, the less favorable state is their dissolution in the very polar region of the membrane/water interface, implying that, in this case, desorption from the membrane into the aqueous phase is the rate-limiting step with an energy barrier $\Delta^{‡}G_{out}^{o}$.

In most studies of solute partition to membranes, the solute is added to the aqueous phase outside unilamellar liposomes. For liposomes with a diameter ≲ 100 nm, the fraction of aqueous media inside the liposomes is negligible, and the final step of equilibration between the inner membrane leaflet and the aqueous medium does not significantly influence the overall solute distribution. In this case, the time needed to reach equilibrium (or the deviation from equilibrium at a given incubation time, $t_{inc}$) depends only on the rate of insertion (with a characteristic time $\tau_{in}=1/k_{in}$) and on the rate of translocation or flip-flop (with a characteristic time $\tau_{f}=1/k_{f}$). For small solutes, the energy barrier for insertion is usually small, and this step is typically completed within seconds at both the lipid concentrations used in vitro and found in vivo [68,106,107,108,109]. Depending on the solute charge and on the strength of the interactions established with the hydrated membrane, the characteristic time for solute translocation between the two membrane leaflets may vary from sub-seconds to several hours [110,111,112,113,114,115,116,117,118,119,120,121], controlling the extent of solute–membrane equilibration. For short incubation times ($t_{inc}$ ≫ $\tau_{in}$, but $\ll \tau_{f}$), equilibration will occur only with the membrane’s outer leaflet. In this case, only the lipid in the outer leaflet should be considered in Equations (1)–(13). In the case of large unilamellar vesicles, this corresponds to half the total lipid, and therefore the fraction of lipid accessible to the solute ($\gamma_{L}$) would be equal to 0.5. On the other extreme, if the system is allowed to equilibrate during at least twice the characteristic time for translocation ($t_{inc}≳2\;\tau_{f}$), almost full equilibration will be achieved and $\gamma_{L}$ ≅ 1.

Partial equilibration leads to an apparent partition coefficient ($K_{P}^{app}$) that underestimates the true $K_{P}$. This is not a major concern when using large unilamellar liposomes where only half of the lipid is in the inaccessible inner leaflet. In this case, an incubation time equal to $\tau_{f}$ leads to $K_{P}^{app}≅0.8\;K_{P}$, and $K_{P}^{app}≅0.55\;K_{P}$ for $t_{inc}={0.1\times \tau }_{f}$. The solute–membrane affinity is thus underestimated by less than a factor of 2.

The underestimation of $K_{P}$ may, however, be very significant when using multilamellar liposomes because the fraction of lipid accessible to the solute added to the outer aqueous media may be very small. As an example, if only 10% of the lipid is in the outer leaflet of the outer lamella, $t_{inc}={0.1\times \tau }_{f}$ leads to $K_{P}^{app}≅0.1\;K_{P}$. Moreover, in this case, equilibration is also dependent on the rate of desorption from the membrane. Incubation for $t_{inc}={2\times \tau }_{f}$ leads to full equilibration with the outer lamella, but depending on the rate of desorption, it may be insufficient for equilibration with the inner lamellae. For very lipophilic solutes, the characteristic time for desorption may reach several hours or even days [5,68,69,71], making incubation until full equilibration an impossible task. It is therefore of high importance to use unilamellar liposomes when characterizing the partition coefficient.

### 2.3. Effect of the Solute on Membrane Properties and Its Own Membrane Affinity

In addition to the electrostatic and kinetic effects discussed in the previous sections, which may be quantitatively corrected, allowing the characterization of the intrinsic solute–membrane affinity, the presence of the solute may induce extensive changes in the membrane properties, leading to a concentration-dependent affinity. Examples of such effects include the interaction of multivalent ions with charged membranes, whose effects on membrane electrostatics are not well described by the Gouy–Chapman theory, may cause lipid condensation, altering the lipid intrinsic curvature and the lateral organization of mixed-lipid membranes, and may induce changes in the topology of the lipidic phase through the promotion of membrane aggregation and fusion [2,122,123,124,125,126,127]; bulk and rigid solutes such as cholesterol, which influence lipid dynamics and membrane lateral organization and solvation properties [128,129,130,131,132,133,134]; or, the case of amphiphilic detergent-like drugs, which, even at relatively low concentrations, may stabilize pores in the membrane and thus lead to concentration-dependent kinetics [44,47,135,136,137,138,139,140]; with several effects being observed for some drugs [44,134,137,138,139,141,142]. While these effects are not expected to be significant in vivo due to the high abundance of membranes and relatively low amount of solute (see discussion on Section 2.1), they may have a significant effect on the solute–membrane affinity obtained in vitro. Common concentration-dependent effects of some specific methodologies are discussed in Section 3, especially for methods that rely specifically on membrane perturbation by the solute, such as changes in the lipid phase transition (Section Methods Based on Changes in the Lipid Phase Transitions) and on membrane saturation with the solute (Section Methods Based on Interactions with Solid-Adsorbed Membranes).

## 3. Methods to Obtain Solute–Membrane Affinity

### 3.1. Experimental Methodologies

There is a large variety of methods that may be used to characterize the association between small molecules and lipid membranes. When the membrane phase is organized as liposomes, if the aqueous media outside the liposomes may be sampled directly, the concentration of solute in this phase may be used to estimate the amount of solute associated with the membrane and thus calculate the partition coefficient. Other approaches are based on changes in the properties of the solute upon association with the membrane, on the changes in the properties of the membranes due to solute association, or on changes in the properties of the whole system. Each approach has advantages and disadvantages, and there are specific formalisms for the analysis of the results to obtain the association affinity. In this section, an extensive review of the different approaches will be done, with the presentation of the formalisms required, limits of applicability, and common pitfalls. The main objective is to provide the framework for a quantitative and critical interpretation of the results reported in the literature for solute–membrane affinity and to facilitate its inclusion in comprehensive databases. It is also intended to guide new researchers in the field in the important task of obtaining accurate estimates for solute–membrane affinity in the case of new or poorly characterized solutes and/or membranes. Some references are provided for each methodology presented, focusing on the early works where the methodologies and analysis formalisms were developed and including also some more recent representative references.

#### 3.1.1. Direct Quantification of the Solute in at Least One of the Phases

**Figure 3:**
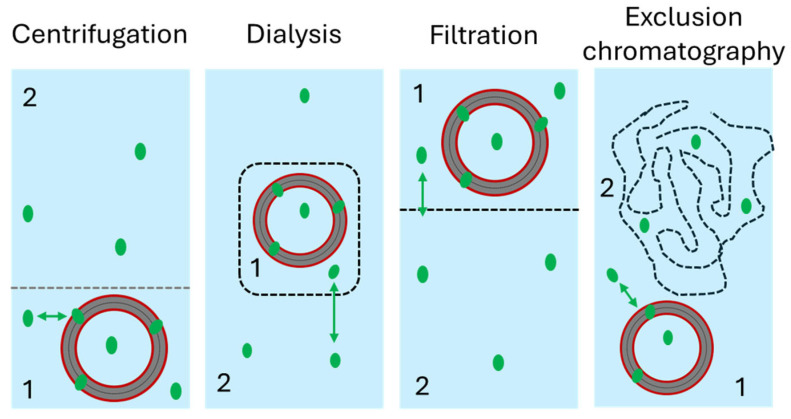
Most usual methods for the physical separation of the aqueous phase (2) and a membrane-enriched solution (1). Membranes are represented as unilamellar vesicles, and the solute (green ellipses) is distributed between the aqueous media and the lipid membrane. The green arrows represent equilibration of the solute in the aqueous media on both sides of the dialysis or filtration membrane and possible desorption of the solute after separation of the vesicles by centrifugation or size exclusion chromatography.

For an accurate quantification of the distribution of solute between the aqueous phase and the membrane, it is better to quantify the amount of solute in both phases, allowing the evaluation of eventual solute degradation and/or adsorption to the apparatus. This procedure cannot, however, be followed to characterize the distribution towards lipid membranes because this phase is organized as liposomes, which always contain large amounts of the aqueous medium. Nevertheless, the aqueous phase may often be physically separated from a membrane-enriched solution, allowing the direct quantification of the solute. The most common methods are schematically represented in Figure 3.

Separation by centrifugation requires a higher density of membranes when compared to the aqueous phase. This approach has the advantage that no separation material is required, avoiding solute loss by adsorption. However, the density of phospholipid membranes is usually only slightly higher than that of pure water, requiring the use of ultracentrifugation for efficient sedimentation of unilamellar vesicles. Also, the presence of salt in the aqueous medium increases the density of this phase, decreasing the density difference or even with the aqueous phase becoming denser than the lipid vesicles. For these reasons, separation by centrifugation is usually followed when the membrane is assembled as multilamellar vesicles [143,144,145,146,147,148,149]. This may, however, lead to an underestimation of the membrane affinity due to incomplete equilibration (see Section 2.2). More recently, unilamellar liposomes encapsulating aqueous media with high density [150,151,152], or with dense particles covered by a phospholipid membrane [153,154,155], have been used instead of MLVs, thus allowing the easy use of centrifugation as the separation method while minimizing the uncertainty in the fraction of lipid accessible.

Equilibrium dialysis permits the physical separation between a membrane-enriched compartment composed of regular unilamellar liposomes and is among the first methods used, still being considered as a reference [48,156,157]. The porous membrane allows equilibration of the solute with the aqueous media outside the dialysis bag while the liposomes are retained. The simplicity of this method justifies its use in most seminal works on solute–membrane interactions [48,156,158,159,160,161,162,163,164,165,166,167]. There are, however, important limitations, including very slow equilibration and a significant adsorption of the solute to the dialysis membrane.

Alternative approaches used to separate the membrane-enriched solution include filtration and ultrafiltration, usually performed in conjunction with centrifugation for a faster separation [144,146,153,154,155,168]. When compared with centrifugation alone, this procedure has the advantage that it does not require vesicles with a higher density than the surrounding aqueous medium. It has, however, the disadvantage of possible adsorption of the solute to the filter, a drawback that is shared with the most established method of equilibrium dialysis.

Size exclusion chromatography is another methodology that could be used to separate the membrane vesicles from the solute in the aqueous medium. This separation methodology has been thoroughly used to evaluate the aggregation behavior of surfactants [169,170], the interaction of solutes with small micelles and proteins [171,172], or the interaction of proteins with liposomes [173]. It has, however, been used only occasionally to evaluate the binding of small molecules to lipid membranes [174], due to the possible removal of solute from the liposomes during elution through the column.

Regardless of the method followed for the physical separation of the aqueous phase and the membrane-enriched solution, the calculation of the partition coefficient should account for the aqueous media contained in the membrane-enriched solution [37] (Equation (19)).

(19) $$K_{P}=\frac{n_{S1}-n_{S2}\frac{V_{W1}}{V_{2}}}{V_{1}-V_{W1}}/\frac{n_{S2}}{V_{2}}$$

The subscript 1 refers to the membrane-enriched solution, and subscript 2 refers to the aqueous phase; $V_{1}$ and $n_{S1}$ are respectively the volume and the mole number of solute in each fraction; and $V_{\mathrm{W1}}$ is the volume of aqueous medium in the membrane-enriched solution. In Equation (19) it is assumed that the aqueous compartment has been efficiently separated from the vesicles and therefore that compartment 2 does not contain membranes.

The volume of aqueous media in the membrane-enriched compartment is relatively small when separation is performed by centrifugation. However, it is very significant when separation is performed by dialysis or filtration, usually several orders of magnitude larger than the volume occupied by the membranes themselves, and the correction introduced by Equation (19) must always be included. An additional common source of artifacts when using dialysis is the increase in volume inside the dialysis container during equilibration, especially if it is a dialysis bag.

The most accurate procedure to calculate the partition coefficient after separation of the two solutions is to quantify the amount of solute on both. However, in some situations only the solute in the aqueous compartment is measured, and the amount of solute in the membrane-enriched phase is calculated from the mass balance. This procedure overlooks eventual adsorption of the solute to the container and filtration material, which may be significant for the case of nonpolar solutes and leads to an overestimation of $K_{P}$. When following this simplified procedure, control experiments should be performed with distinct amounts of lipid vesicles to evaluate for consistency in the calculated partition coefficient.

The assumption that the aqueous phase does not contain lipids may also not hold, especially when the lipids tend to associate as small micelles, as is the case for lysophospholipids and fatty acids. The presence of residual amounts of lipid in the aqueous phase may significantly increase the amount of solute in the “membrane-free” compartment, leading to an underestimation of $K_{P}$. This is particularly relevant for solutes with very large affinities for the membranes.

In addition to the methods indicated above, where the quantification of the solute in the aqueous phase requires physical separation from a membrane-enriched solution, there are a few methods where it may be performed with the membranes dispersed in the aqueous phase. One example is the case of volatile solutes, where their vapor pressure above the sample provides an accurate measure of the concentration in the aqueous phase. This approach has been followed to obtain the partition coefficient of ethanol to membranes of distinct lipid compositions, with the concentration of ethanol being quantified by headspace gas chromatography [175]. Other methods take advantage of a second equilibrium involving the solute in the aqueous phase. This is the case for the association of fatty acids or lysolipids with the fluorescent fatty acid binding protein ADIFAB [176,177,178,179,180,181,182,183,184]. In this approach, it must be verified if the association of the solute to the reporter binding agent does not displace the solute from association with the membranes. Otherwise, the two equilibria must be considered when calculating the solute–membrane affinity.

#### 3.1.2. Indirect Quantification of the Solute from Changes in Its Properties

When an easily measurable property of the solute changes upon association with the membranes, this may be used to indirectly quantify the amount of solute in the membranes. This approach has been followed by a large diversity of methods based on changes of very distinct properties. The major advantage in comparison with the methodologies presented in Section 3.1.1 is that the equilibrium of the solute between the aqueous phase and the membranes is not disturbed by the physical separation of the two phases. The major disadvantage is that it is an indirect measurement, and therefore some general methodological aspects must be taken into consideration.

The Master Equation, when following the variation in a solute’s property, is given in Equation (20):

(20) $$P_{S}=f_{S_{W}}P_{S_{W}}+f_{S_{M}}P_{S_{M}}$$

where $P_{S}$ is the property of the solute measured at a given lipid concentration, $P_{S_{W}}$ is the same property when all solute is in the aqueous phase, and $P_{S_{M}}$ when the solute is associated with the membrane phase; $f_{S_{W}}$ and $f_{S_{M}}$ correspond to the fraction of solute in the aqueous phase and associated with the membrane, respectively. The fraction of solute in each phase is dependent on the amount of membrane in solution and on the equilibrium association parameter, which may be defined in different ways as described in Section 1.

The validity of Equation (20) relies on several aspects that should be verified:

1. The fraction of the membrane accessible to the solute during the equilibration time must always be considered. As indicated in Section 2.2, if the membrane is organized as unilamellar vesicles, this introduces a relatively small uncertainty (up to a two-fold underestimation of the affinity) but corresponds to a major uncertainty if multilamellar vesicles are used.
2. It is assumed that the property of interest for the solute in the aqueous media and associated with the membrane is not dependent on the lipid concentration nor on the local concentration of solute; that is $P_{S_{M}}$ and $P_{S_{W}}$ are constants in the concentration range characterized. This is a major requirement and is not always guaranteed. Frequent problems are due to attaining very high local concentrations of solute in the membrane. This may lead to changes in the properties of the membranes, to alterations in the position of the solute in the membrane, and in the interactions established. An indication regarding the validity of this assumption may be obtained by calculating the lipid/solute ratio considering the partition coefficient obtained from the best fit to the changed property. For small and uncharged solutes, a ratio larger than 20 at all lipid concentrations tested is a good indication that the membrane remains unperturbed [97,185]. An exception is when fluorescence is the property being followed. In this case, the ratio should always be higher than 100 due to possible fluorescence self-quenching even at solute concentrations as low as 1 mol% [186,187,188]. A higher lipid/solute ratio should also be used for the case of large solutes or when their global charge is very high, because each solute molecule influences the properties of many lipids or leads to a strong variation in the membrane electrostatic properties, respectively. Another common problem when using very hydrophobic solutes is the use of solute concentrations above their critical aggregation concentration in the aqueous media. In this case, there are two equilibria in the system, and if the aggregation equilibrium in the aqueous phase is not included in the analysis, it will lead to an underestimation of the partition coefficient. The contribution from this problem may be evaluated through performing experiments at different solute concentrations, and/or by following the time evolution of the property at a given lipid concentration (see e.g., [5,189]).
3. The properties of the solute when associated with the membranes ($P_{S_{M}}$) must be well characterized. This requires evaluating the property for a fractional volume of membrane above $1/K_{P}$, where most of the solute is associated with the membrane. For solutes with small affinities for the membranes ($K_{P}\leq 10^{2}$) this requires going to very high lipid concentrations ($\left[L\right]\gg 10\;\mathrm{mM}$). The upper limit of lipid concentration with the lipid phase organized as unilamellar vesicles of 100 nm diameter prepared by extrusion is around 100 mM (corresponding to 1/3 of the suspension volume being occupied by the LUVs), and those high concentrations can only be achieved with charged membranes to decrease the tendency to form multilamellar vesicles. The use of liposomes to characterize solute–membrane affinity is therefore limited to solutes with $K_{P}>10$. At these high lipid concentrations, the solution presents high turbidity and may lead to severe artifacts when an optical property is followed. Corrections for background signals do not always solve the problem because the contributions may be non-additive. One possible approach to evaluate the magnitude of those effects is to use distinct excitation and/or emission pathlengths and check for consistency. Alternatively, the effects may be corrected through control experiments using solutes that have been previously well characterized (e.g., [190]).

In the following sections, the most common methodologies to obtain the partition coefficient from changes in the properties of the solute are presented, with focus on method-specific aspects that are not thoroughly addressed in available reviews.

##### Quantification of the Solute from Changes in Its UV-Vis Spectroscopic Properties

UV-vis absorption is a convenient methodology to follow the transfer of the solute between the aqueous and the membrane phases, given the easy availability of the required equipment and the high absorptivity of most bioactive molecules, at least in the UV region. However, this property depends weakly on the environmental properties, and most molecules show little variation upon association with the membrane. Another important drawback when following absorption in the UV region is the strong light scattering by the membrane vesicles. These limitations are somewhat overcome when using derivative spectrophotometry [191,192], and this approach has been successfully used to characterize the partition coefficient of several molecules [3,191,192,193,194,195,196,197,198,199,200,201,202]. To avoid artifacts introduced by non-additivity of the signals due to saturation, it is highly recommended that the equipment baseline be performed with a non-absorbing solution, with the changes in the absorption of the solute due to interaction with the membrane being obtained by subtracting the absorption from the blank solutions (containing all components except the solute). When the experiments are performed in the absence of signal saturation (e.g., using a low- to moderate-concentration range of lipid membrane), the second derivative numerical method straightforwardly eliminates the contribution from light scattering by the liposome suspensions, since the background scatter is nearly described by a linear function of the wavelength whose second derivative is zero [198].

In contrast with UV-vis absorption, fluorescence emission is a property that is strongly dependent on the environment. The polarity and viscosity of the lipid membrane differ significantly from that of the aqueous medium, and, therefore, the fluorescence properties of the solute usually change upon association with the membrane. Those properties are very convenient to evaluate the solute–membrane association due to the high sensitivity and low interference from other compounds present in the system. This approach is particularly adequate to characterize fluorescent probes due to their high fluorescence quantum yield. Nevertheless, a large fraction of bioactive molecules has aromatic groups in their structure, providing them with some fluorescence at least in the UV region.

The most commonly followed property is the variation in the fluorescence quantum yield and/or spectra [16,69,71,107,144,190,203,204,205,206,207,208,209,210,211,212,213,214], although changes in fluorescence lifetime have also been used [205,215,216]. The fluorescence quantum yield and lifetime are also influenced by the presence of other solutes in the system, and this has been used to follow the partition of the solute to the membrane. An example is the variation of the fluorescence intensity due to quenching and/or fluorescence resonance energy transfer occurring only in one of the phases [16,204,209,217,218,219,220,221]. In addition to providing information regarding the fraction of solute in each phase, this approach may also give valuable information regarding the transversal location of the solute in the membrane [204,217,222,223,224,225].

Due to the higher viscosity of the membrane when compared to the aqueous medium, the fluorescence anisotropy of the solute usually increases when associated with the membrane. This property is therefore also commonly used to follow solute–membrane association [204,205,206,226]. An important advantage of this approach is that it does not depend on solute concentration, at least if it is maintained low enough to prevent significant depolarization due to homo-FRET in the case of overlapping absorption and emission spectra [227,228]. It is, however, strongly influenced by scattered light, which represents an important drawback if using large vesicles and fluorescence in the UV region.

Fluorescence correlation spectroscopy (FCS) is another methodology that may be used to follow solute association with the membrane, relying on the much smaller diffusion coefficient of the large liposomes [152,229].

**Figure 4:**
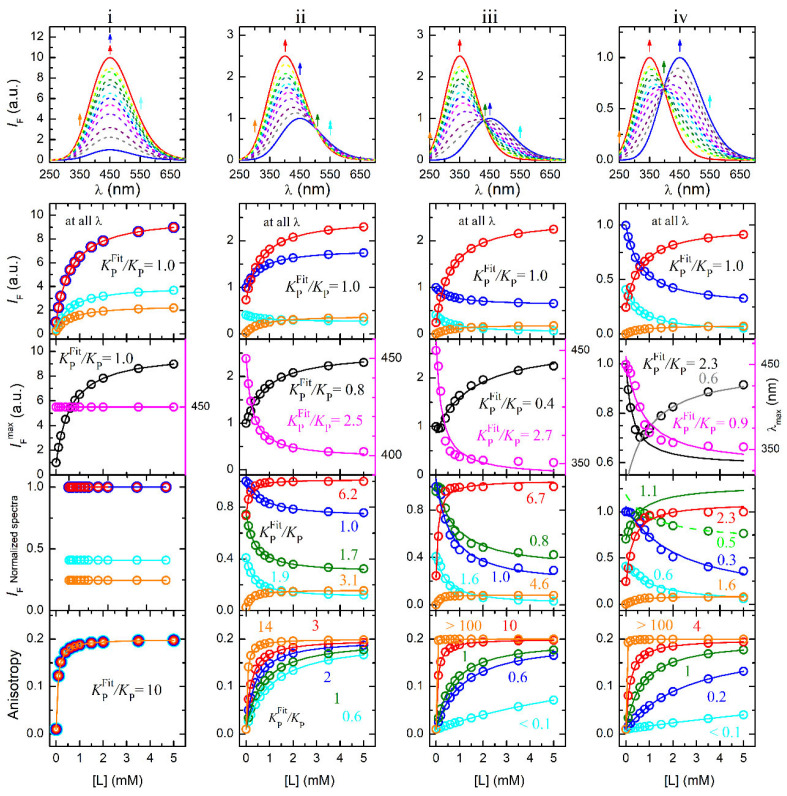
Effect of the lipid concentration on the fluorescence properties of a solute and corresponding partition coefficient ($K_{P}^{Fit}$) obtained from the best fit of the Master Equation (20). The fluorescence spectra were simulated by a LogNormal function with s = 0.15, varying the wavelength of maximum emission and fluorescence quantum yield for the solute in the aqueous media ($\lambda_{W}$ and $\phi_{W}$, respectively) and associated with the membrane ($\lambda_{M}$ and $\phi_{M}$, respectively). Case (**i**): $\lambda_{W}=\lambda_{M}=$ 450 nm, and $\phi_{M}/\phi_{W}=$ 10; Case (**ii**): $\lambda_{W}=$ 450 nm, $\lambda_{M}=$ 400 nm and $\phi_{M}/\phi_{W}=$ 2.5; Case (**iii**): $\lambda_{W}=$ 450 nm, $\lambda_{M}=$ 350 nm and $\phi_{M}/\phi_{W}=$ 2.5; and Case (**iv**): $\lambda_{W}=$ 450 nm, $\lambda_{M}=$ 350 nm and $\phi_{M}/\phi_{W}=$ 1. **Top plots** show the spectra of the solute in the aqueous phase (**─**) and in the membrane (─), and the dashed lines correspond to the spectra at distinct lipid concentrations obtained from the pure spectra and the fraction of solute in the aqueous phase and membrane predicted from Equation (6) for a partition coefficient ($K_{P}$) of 2 × 10^3^ and a lipid volume of 0.8 dm^3^/mol. The arrows indicate the emission wavelengths at which the fluorescence properties were obtained, corresponding to $\lambda_{M}$-100 (↑), $\lambda_{M}$ (↑), $\lambda_{W}$ (↑), $\lambda_{W}$+100 (↑), and the isoemissive emission wavelength (↑).The other plots show the dependence of the fluorescence properties with the lipid concentration and the ratio of $K_{P}^{Fit}$ obtained from the best fit and the true $K_{P}$ using the color code indicated by the arrows. The properties considered are the fluorescence intensity at a given fixed wavelength ($I_{F}$); the maximum fluorescence intensity ($I_{F}^{max}$) and the wavelength at which the maximum fluorescence intensity is observed ($\lambda_{max}$); the fluorescence intensity at a given fixed wavelength after normalization of the spectra at $I_{F}^{max}$ ($I_{F}Normalized$); and the fluorescence anisotropy at a given emission wavelength considering $r_{W}$ = 0.01 and $r_{M}$ = 0.2.

There are several reviews in the literature where the specificities of each fluorescence-based approach are described in detail [42,43,227,228,230], and they will not be addressed in this review. The focus of this review is to complement the information available with a discussion on the validity limits of the Master Equation (20), namely regarding the additivity of the contribution from the solute in each phase. With this goal, the results expected in some typical situations were simulated. The dependence of several fluorescence properties on the lipid concentration and the partition coefficient obtained from the best fit of the Master Equation are shown in Figure 4. The variable properties considered were the fluorescence quantum yield ($\phi_{F}$) and the wavelength at maximum fluorescence emission ($\lambda_{max}$). The conditions simulated are (i) a large (10-fold) increase in $\phi_{F}$ upon partition to the membrane without changes in $\lambda_{max}$; (ii) a moderate (2.5 fold) increase in $\phi_{F}$ accompanied by a moderate variation in $\lambda_{max}$ (50 nm blue-shift); (iii) a moderate variation in $\phi_{F}$ (2.5-fold) and large variation $\lambda_{max}$ (100 nm blue-shift); and (iv) no variation in $\phi_{F}$ and large variation $\lambda_{max}$ (100 nm blue-shift). For simplicity, it was assumed that the solute absorption spectrum (shape and molar absorptivity) is the same when in the aqueous or membrane phases. It is also assumed that the solute does not change its ionization state upon association with the membranes, which means that no additional equilibria must be considered other than solute equilibration between the aqueous and membrane phases. The ratio between the partition coefficient estimated from the best fit of the simple Master Equation ($K_{P}^{Fit}$), with $f_{S_{M}}$ calculated from Equation (6), and the true $K_{P}$ (fixed at 2 × 10^3^), is included in Figure 4. A manuscript is in preparation describing in detail the simulation methodology and the interpretation of the results obtained for a broader range of hypothetical conditions, including the incorporation of experimental data to assess the membrane affinity of selected fluorescent drugs and probes.

The first observation is that the dependence of the fluorescence property on the lipid concentration is usually well described by the Master Equation (20) for all properties evaluated. However, the value of $K_{P}^{Fit}$ obtained from the best fit may deviate from the true $K_{P}$ by more than an order of magnitude. Therefore, the quality of the best fit provides no evidence regarding the validity of the approximations assumed in the derivation of the Master Equation.

The first property analyzed was the fluorescence intensity at a fixed wavelength ($I_{F}$); the first set of plots below the spectra. This is a very robust property, with $K_{P}^{Fit}$ being always equal to the true $K_{P}$ irrespective of the wavelength selected.

The adequacy of the maximum fluorescence intensity ($I_{F}^{max}$) was also evaluated (black curves in the middle plots). In the absence of a spectral shift, this is equivalent to the intensity at a fixed wavelength, leading to $K_{P}^{Fit}=K_{P}$. However, as the spectral shift becomes significant, the overlap between the spectra of the solute in the aqueous media and the membrane decreases, and this property leads to incorrect estimations of the partition coefficient. If the spectra overlap is very small (as in case iv), the shape of the variation is not well described by the Master Equation, with the results at low and high lipid concentrations leading to different $K_{P}^{Fit}$.

The selection of intensive fluorescence properties may be of high value to characterize the solute–membrane affinity using high-throughput methodologies where solute concentration may show some variations along the distinct datapoints. In the case of significant spectral shift, a convenient property could be the wavelength of maximum fluorescence intensity ($\lambda_{max}$). The dependence of $\lambda_{max}$ is shown in the middle plots (magenta curves). For small spectral shifts maintaining a large overlap between the emission spectra of the solute in water and in the membrane (case ii), a reasonably good fit is obtained but $K_{P}^{Fit}$ leads to an overestimation of $K_{P}$. This may, however, be corrected if weighed by the relative fluorescence intensity of the solute in both media. In this case $I_{F}^{max}\left(S_{M}\right)/I_{F}^{max}\left(S_{W}\right)$ = 2.5, and an accurate estimate of $K_{P}$ is obtained when $K_{P}^{Fit}$ is divided by this ratio. However, as the spectral shift increases (cases iii and iv), poor fits are obtained. In the simulations shown, considering lipid concentrations that lead up to 90% of the solute in the membrane, although the quality of the best fit is not good, $K_{P}^{Fit}$ leads to a reasonable estimate of $K_{P}$ when divided by the relative intensities in both media ($I_{F}^{max}\left(S_{M}\right)/I_{F}^{max}\left(S_{W}\right)$ = 2.5 for case iii and 1.0 for case iv). However, in a real situation, the poor quality of the best fit will lead to $K_{P}^{Fit}$ values that vary with the lipid concentration used, showing that this property is not robust enough for the estimation of partition coefficients.

In the second plots from the bottom, the variation of the signal at a given wavelength is represented after normalization of the different spectra at their own maxima, $I_{F}\;Normalized\;spectra$, with the color codes corresponding to the emission wavelengths indicated by the arrows in the top plots. As expected, no variation is observed in the absence of a spectral shift (case i). For a small spectral shift and moderate increase in the fluorescence quantum yield (case ii), the shape of the curve is well described by the Master Equation at all wavelengths. However, the best fit leads to incorrect estimations of $K_{P}$, usually leading to an overestimation. A good agreement was obtained when the wavelength of maximum emission in the aqueous phase was selected (blue curves), but this adequacy is not robust enough to be used for the characterization of unknown partition coefficients. As the spectral shift increases (cases iii and iv), the curve shape is no longer well described by the Master Equation. In the case of a large spectral shift without changes in quantum yield (case iv), selecting the isoemissive wavelength reveals two distinct regimes: an increase in normalized fluorescence intensity at low lipid concentrations and a decrease at high lipid concentrations. Moreover, each regime leads to a different fitted partition coefficient (green dark solid line and light green dashed line).

Fluorescence anisotropy is another intensive parameter that was evaluated in Figure 4 (lower plots). The shape of the curve is always very well described by the Master Equation. However, the value obtained for $K_{P}^{Fit}$ deviates significantly from $K_{P}$ at some of the conditions considered. The inadequacy of variations in the fluorescence anisotropy as a direct reporter of solute partition to the membrane has been clearly stated before (e.g., [42]) and is due to the fact that the contribution of the distinct solute species (in the aqueous or membrane compartment) to the overall parameter is not proportional to their fraction. This may be corrected by weighting the fraction of the species by their contribution ($\alpha_{W}$ or $\alpha_{M}$), Equation (21), [42,205,231].

(21) $$P_{S}=\frac{\alpha_{W}{\;f}_{S_{W}}P_{S_{W}}+\alpha_{M}{\;f}_{S_{M}}P_{S_{M}}}{\alpha_{W}{\;f}_{S_{W}}+\alpha_{M}{\;f}_{S_{M}}}$$

The contribution of each species depends on the intensity of light absorbed, which is proportional to their molar absorptivity at the excitation wavelength ($\varepsilon_{\lambda \mathrm{exc}}^{S_{x}}$) if absorption is lower than 0.1, and on their fluorescence emission intensity at the emission wavelength considered, which depends on the fluorescence quantum yield ($\phi_{F}^{S_{x}}$) and on the fraction of emitted light at the collected wavelength ($g_{\lambda \mathrm{em}}^{S_{x}}$) corresponding to the fluorescence intensity at that wavelength on a fluorescence spectrum normalized by its area (Equation (22)).

(22) $$\alpha_{W}=\varepsilon_{\lambda \mathrm{exc}}^{S_{W}}\;\phi_{F}^{S_{W}}\;g_{\lambda \mathrm{em}}^{S_{W}}\;;\;\alpha_{M}=\varepsilon_{\lambda \mathrm{exc}}^{S_{M}}\;\phi_{F}^{S_{M}}\;g_{\lambda \mathrm{em}}^{S_{M}}$$

When the anisotropy obtained from the simulated results is analyzed with the equation resulting from (21) and (22), the value of $K_{P}^{Fit}$ is essentially equal to $K_{P}$, reflecting the fact that anisotropy is an additive property if weighted by the fractional fluorescence intensity from each species [232]. In contrast, the related property fluorescence polarization is not additive even when weighted by the fractional fluorescence intensity [232], and it cannot, therefore, be used to obtain the affinity of the solute to the membrane.

The very large number of parameters in Equation (21) may discourage the best-intentioned researcher and may justify the inadequate use of the simple Master Equation in many publications. It should, however, be noted that the individual parameters do not need to be known, only the ratio when the solute is in the membrane and in water ($\alpha_{M}/\alpha_{W}$). These parameters may be easily obtained through the relative fluorescence intensity in the aqueous phase and in the presence of sufficiently high amounts of the membrane phase, permitting the use of the correct formalism without too much effort (as done e.g., in [233]). When the fluorescence intensity from both species is similar at the wavelength selected (namely at the isoemissive wavelength, shown in green), $K_{P}$ may in fact be obtained directly from the best fit of Equation (20).

**Figure 5:**
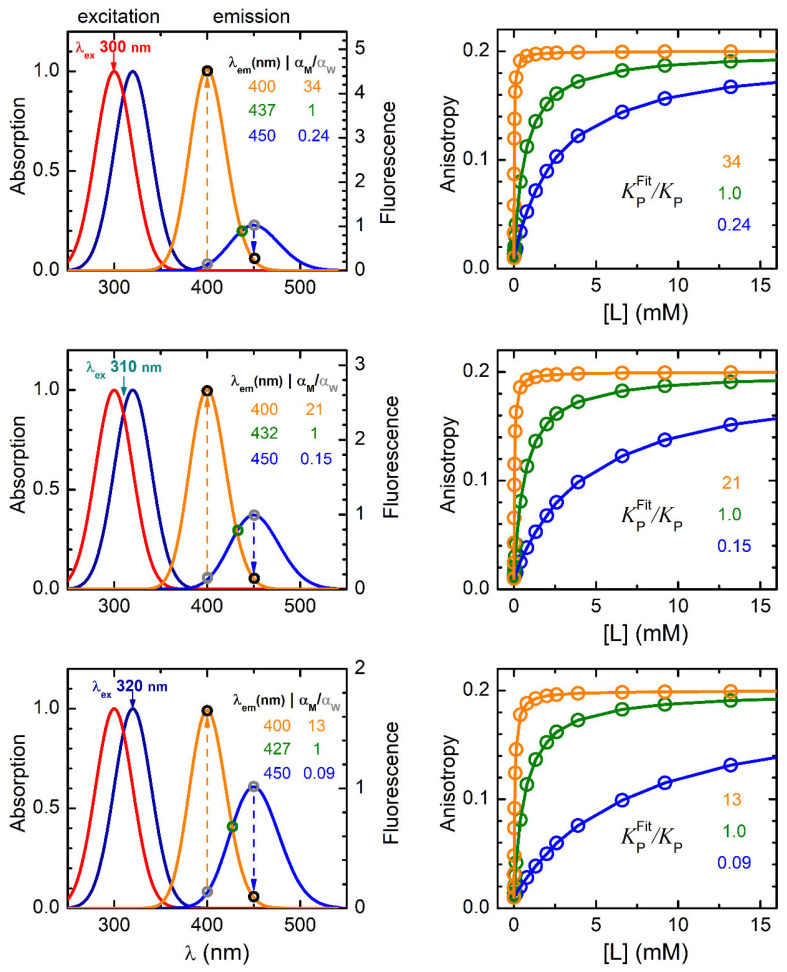
Effect of the excitation ($\lambda_{ex}$) and emission ($\lambda_{em}$) wavelengths on the apparent partition coefficient obtained when analyzing the dependence of the fluorescence anisotropy on the lipid concentration using the Master Equation. **Left plots**: Absorption and fluorescence emission spectra of the species when in the aqueous phase (**—** and **—**, respectively) and when associated with the membrane (**—** and **—**, respectively), both normalized by the maximum of the species in water. **Right plots**: Variation of the fluorescence anisotropy with the lipid concentration for excitation at the wavelength indicated in the left plot and fluorescence emission collected at three distinct wavelengths with the color code indicated in the left plot. The ratio $\alpha_{M}/\alpha_{W}$ defined by Equation (22) at each condition is shown in the left plots, and the ratio between the apparent partition coefficient obtained from the best fit of Equation (20) and the true $K_{P}$ is indicated in the right plots with the corresponding color code. The simulations were performed assuming $K_{P}$ = 2 × 10^3^, $\Phi_{F}^{M}/\Phi_{F}^{W}$ = 2, a maximum absorption at 320 nm and 300 nm, and a maximum fluorescence emission at 450 nm and 400 nm for the species in water and associated with the membrane, respectively.

In Figure 4, it was assumed that the absorption spectrum is the same in both media. The effect of changing the excitation wavelength when there are variations in the absorption spectra is explored in Figure 5. A common situation was considered where the absorption and the emission spectra are shifted to higher energies (lower wavelengths) when the polarity of the medium decreases ($\lambda_{maxA}^{W}$ = 320 nm and $\lambda_{maxA}^{M}$ = 300 nm, and $\lambda_{maxF}^{W}$ = 450 nm and $\lambda_{maxF}^{M}$ = 400 nm). It was also assumed that the molar absorptivity at the maximum absorption was unchanged, while a higher fluorescence quantum yield was considered when associated with the membrane ($\Phi_{F}^{M}/\Phi_{F}^{W}$ = 2), the anisotropy was the same as in the simulations shown in Figure 4, $r_{W}$ = 0.01 and $r_{M}$ = 0.2.

The first observation from Figure 5 is that if the combination of the excitation and emission wavelengths favors the species in the aqueous media, the apparent partition coefficient obtained when the anisotropy variation is fitted with the unweighted Equation (20) is lower than $K_{P}$ (wavelengths and anisotropies marked in blue). Conversely, if the species in the membrane are favored then $K_{P}^{Fit}>K_{P}$ (wavelengths and anisotropies marked in orange). On the other hand, if the fluorescence emission is collected at a wavelength where the fluorescence intensity is similar when the solute is in the water or associated with the membrane, $\alpha_{M}/\alpha_{W}≅\;$1, a good estimate of $K_{P}$ is obtained from the fit of the unweighted equation (wavelengths and anisotropy variations marked in green). The wavelength where this is observed depends on the excitation wavelength—closer to $\lambda_{maxF}^{W}$ at excitation wavelengths that preferentially excite the solute in the membrane (bottom-to-top plots). Therefore, even if the absorption spectra, molar absorptivities and/or fluorescence quantum yields are unknown, an accurate estimation of $K_{P}$ may be obtained provided that the fluorescence emission is collected at a wavelength where both species show similar fluorescence intensity.

In any case, $K_{P}$ may always be calculated from $K_{P}^{app}$ provided that $\alpha_{M}/\alpha_{W}$ is known (Equation (23)), which is obtained from the equality of Equations (24) and (25).

(23) $$K_{P}^{Fit}=K_{P}\frac{\alpha_{M}}{\alpha_{W}}$$

(24) $$r=\frac{\alpha_{W}f_{W}\;r_{W}+\alpha_{M}\;f_{M}\;r_{M}}{\alpha_{W}f_{W}+\alpha_{M}\;f_{M}},\;\mathrm{with}\;f_{W}=\frac{1}{V_{W}+K_{P}V_{M}}\;\mathrm{and}\;f_{M}=\frac{K_{P}V_{M}}{V_{W}+K_{P}V_{M}}$$

(25) $$r=f_{W}^{Fit}\;r_{W}+f_{M}^{Fit}\;r_{M},\;\mathrm{with}\;f_{W}^{Fit}=\frac{1}{V_{W}+K_{P}^{Fit}V_{M}}\;\mathrm{and}\;f_{M}^{app}=\frac{K_{P}^{Fit}V_{M}}{V_{W}+K_{P}^{Fit}V_{M}}$$

In conclusion, the best property to report on the association of fluorescent solutes to membranes is the fluorescence intensity at a fixed wavelength. This property is robust and provides good direct estimates in all cases. If the conditions of the experiment introduce variability in the concentration of solute, fluorescence anisotropy may be used, but it must be weighted by the relative fluorescence intensity of the solute in the distinct phases. Unfortunately, simple corrections with general validity cannot be applied to the convenient properties of spectral shift or $I_{F}\;Normalized\;spectra$ to estimate the true $K_{P}$. Spectral shifts may be used but only if the spectra of the solute in water and in the membrane strongly overlap (case ii in Figure 4). In this case a good fit of the Master Equation is expected, and the true $K_{P}$ may be obtained from Equation (23). These properties should therefore be used with caution, preferably supported by alternative independent approaches.

##### Quantification of the Solute from Changes in Its Ionization Equilibria

The variation of solute ionization equilibria is a property that has also been used often to characterize the association of weak acids and bases with membranes [48,165,234,235,236,237,238,239]. This may be obtained directly by potentiometric titration or through variations in the solute properties at different pH values in the presence of varying concentrations of lipid.

**Figure 6:**
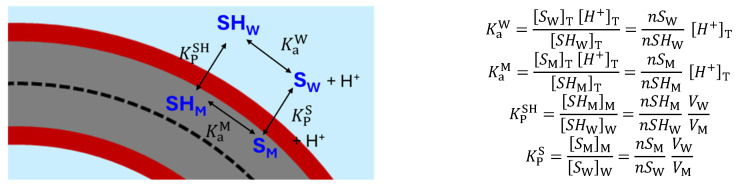
Interaction of an ionizable species with lipid membranes showing the equilibrium of ionization when in the aqueous medium and associated with the membrane ($K_{a}^{W}$ and $K_{a}^{M}$, respectively) and partition of the protonated and deprotonated species ($K_{P}^{SH}$ and $K_{P}^{S}$, respectively). The subscript in each concentration term refers to the volume considered to calculate the concentration, being M or W when considering only the volume of the membrane or aqueous phase and T if the total volume of the solution is considered.

The equilibria that must be considered include the ionization of the solute in the aqueous phase and when associated with the membrane, as well as the partition coefficient of all solute species for the membrane. In the case of solutes with a single ionization equilibrium, the relevant equilibria are shown in Figure 6.

The four equilibria establish a thermodynamic cycle, so the equilibrium constants are constrained by the following relation:

(26) $$K_{P}^{\mathrm{SH}}{\;K}_{a}^{M}=K_{P}^{S}{\;K}_{a}^{W}\;;\;pK_{a}^{M}=pK_{a}^{W}-log\left(\frac{K_{P}^{S}}{K_{P}^{\mathrm{SH}}}\right)$$

At a given pH and volume of membrane, the solute is distributed between the distinct species and compartments, showing a global, observed, ionization constant ($K_{a}^{obs}$) given by the following:

(27) $$K_{a}^{obs}=\frac{{nS}_{W}+{nS}_{M}}{{nSH}_{W}+{nSH}_{M}}{\;\left[H^{+}\right]}_{T};pK_{a}^{\mathrm{obs}}=pK_{a}^{W}+log\left(\frac{1+K_{P}^{\mathrm{SH}}\nu }{1+K_{P}^{S}\nu }\right)$$

where $\nu =V_{M}/V_{W}≅V_{M}/V_{T}$, and the observed, global, partition coefficient ($K_{P}^{\mathrm{obs}}$) is given by the following:

(28) $$K_{P}^{\mathrm{obs}}=\frac{\left({\;\left[H^{+}\right]}_{T}+K_{a}^{M}\right)K_{P}^{\mathrm{SH}}}{\left({\;\left[H^{+}\right]}_{T}+K_{a}^{W}\right)}$$

**Figure 7:**
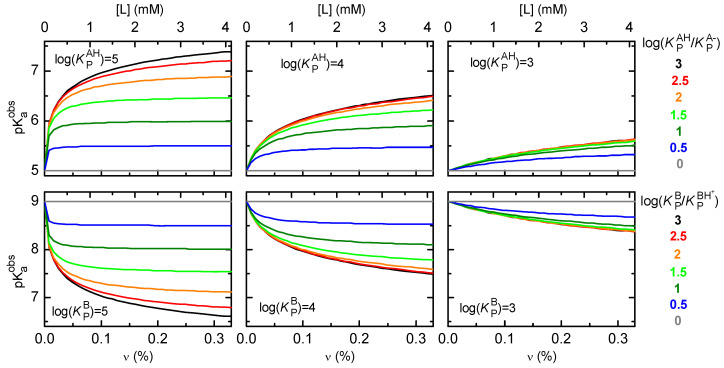
Effect of the lipid concentration and resulting ratio of the membrane and water phase volumes on the solute $pK_{a}^{\mathrm{obs}}$, in the case of acids ($pK_{a}^{W}\;$ = 5, **upper plots**) and bases ($pK_{a}^{W}\;$ = 9, **lower plots**), for solutes with different partition coefficients of the neutral form (**plots left** to **right**) and different relative partition coefficients of the neutral and ionized forms (distinct curves in each plot, color code defined on the right).

Simulations for the dependence of $pK_{a}^{\mathrm{obs}}$ on ν for some typical situations are shown in Figure 7.

The different lines in each plot correspond to different magnitudes of stabilization of the partition coefficient of the neutral species (AH or B, for acids and bases, respectively). In the case of equal membrane affinities ($K_{P}^{AH}=K_{P}^{A^{-}}$, or $K_{P}^{B}=K_{P}^{{BH}^{+}}$), the ionization equilibrium is unaffected by partition to the membrane, and no variation is observed in the $pK_{a}^{\mathrm{obs}}$ (grey lines). Stabilization of the neutral species by the membrane leads to an increase in its fraction at higher lipid concentrations, leading to an increase in $pK_{a}^{\mathrm{obs}}$ in the case of acids (upper plots) and a decrease in the case of bases (lower plots). The effect of the lipid concentration on $pK_{a}^{\mathrm{obs}}$ depends on the lipophilicity of the solute, with less than one $pK_{a}$ unit at 4 mM lipid for solutes with moderate lipophilicity ($logK_{P}^{AH}$ = 3 or $logK_{P}^{B}$ = 3, right plots) and up to several $pK_{a}$ units for very lipophilic solutes ($logK_{P}^{AH}$ = 5 or $logK_{P}^{B}$ = 5, left plots).

One difficulty of using potentiometric titration to characterize the interaction of ionizable solutes with membranes is the high concentrations of solute that must be used to allow a direct pH titration. This leads to very high local concentrations in the membrane that may alter the surface electrostatic potential of the membrane and influence the affinity observed. Corrections for electrostatic effects are therefore usually required to characterize the association of the charged solute species with the membranes. This is achieved by substituting the partition coefficients in Equation (27) with the intrinsic partition coefficients that depend on the electrostatic surface potential of the membrane; see Equations (14)–(18). This was performed in reference [237] to obtain the intrinsic partition coefficients of several weak acids and bases. Some additional challenges with the application of this methodology include the presence of ionizable groups in the lipids themselves and the possible displacement of the ionization equilibria in the aqueous media at the water/membrane interface due to accumulation or depletion of $H^{+}$ and/or ionized solute species (see e.g., reference [48] for details).

Several additional methodologies may be used to indirectly follow solute ionization in the presence of the membrane and thus obtain the partition coefficient of the distinct species and $pK_{a}^{M}$. This may be achieved by obtaining $K_{P}^{obs}$ at distinct pH values or through the characterization of $pK_{a}^{obs}$ at distinct lipid concentrations. The only requirement is that the methodology be able to distinguish between the different solute ionization species when in the aqueous medium and associated with the membrane. All concerns discussed in the preceding sections will apply, namely the effects of the solute on membrane properties, effects of the interaction kinetics, and liposome multilamellarity (discussed in Section 1), as well as issues with additivity of the measured property (as discussed in the preciding subsection).

Isothermal titration calorimetry (ITC) is of particular relevance to characterize partition and ionization and will be discussed in detail in its respective section below. Fluorescence-based methodologies are also useful since ionization usually influences the fluorescence spectra and/or quantum yield and because their high sensitivity allows the use of very low solute concentrations, providing the parameters in unperturbed membranes. However, this methodology is difficult to implement due to changes in the fluorescence properties of the solute when associated with the nonpolar membrane, the full characterization requiring knowledge of the absorption and fluorescence spectra of all species in the aqueous medium and associated with the membrane [233]. Other relevant methodologies are briefly discussed in the next section.

The characterization of the partition and ionization equilibria for ionizable solutes is of major practical importance when the goal is to predict the behavior of the solutes in vivo, namely their absorption, distribution, metabolism, elimination, and toxicity (ADME/Tox) as well as their pharmacodynamics. This is because all those processes are influenced by the localization of the solute in the complex in vivo system (namely, the aqueous compartments and biomembranes), and because the pH varies in the distinct aqueous compartments of the cells, tissues, and organs. The charge of the solute when associated with the biomembranes has a strong influence on its rate of membrane permeation (with direct effects on its pharmacokinetics) and on its transversal location in the membrane (influencing its interaction with membrane proteins and therefore its pharmacodynamics). Due to its complexity, there are, however, a very limited number of studies where the interplay between solute ionization and membrane association has been characterized in detail. Hopefully, the computational tools more recently available will facilitate the analysis and the interpretation of complex sets of data and will contribute to overcoming these limitations.

##### Other Methodologies Based on Changes in Solute Properties

Nuclear magnetic resonance (NMR) is a powerful technique that has been used to study solute–membrane interactions. This is a powerful and versatile methodology where the resonance may be followed from distinct nuclei (most commonly ^1^H, ^2^H, ^13^C, ^31^P, ^19^F), in solution and solid-state NMR, with many variations in the pulse sequences used for the spectra acquisition including, 1-dimensional and multi-dimensional NMR. A major advantage of this methodology is the possibility of obtaining the localization of the solute in the membrane [240,241,242]. Effects of the solute on membrane properties can also be obtained, including changes in the lipid order (e.g., [24]) or membrane curvature (e.g., [243]) due to intercalation of the solute, as well as surface electrostatics due to changes in the head group density or association of charged solutes (e.g., [244]). The resonances from the solute nuclei are not only sensitive to the polarity of the environment but also to changes in ionization, thus allowing the characterization of changes in pKa upon association with the membrane [245]. In some conditions, it is possible to distinguish between the solute associated with the outer and inner leaflets of the membrane, thus allowing the characterization of the rate of permeation [246,247]. Information about the rate of solute equilibration with the membrane can also be obtained through the dependence of the solute resonance frequency and width on the lipid concentration, with slow equilibration originating resonances at two distinct frequencies for the solute in water and in the membrane [248,249,250,251], while in fast equilibration, resonance at a single intermediate frequency is obtained [247,251,252,253,254,255,256].

In spite of the immense potential, there are very few reports on the use of NMR to obtain partition coefficients [249,253,254,255,256,257,258], most using ^19^F [249,254,255] but also ^1^H [253,256,258] and ^2^H [250,257]. In most of the publications, only qualitative or semi-quantitative information is obtained [259,260,261,262,263,264,265,266]. The major difficulty is signal broadening due to the slow tumbling of the large lipid aggregates. This is overcome through the use of small lipid aggregates such as small unilamellar vesicles (SUVs) [253,255] or lipid-surfactant bicelles [258], by following only the unbound solute [254,256], or through the use of large lipid vesicles in solid-state NMR with fast spinning [249]. All strategies have their limitations, with the small lipid aggregates being poor model systems for biomembranes, the existence of non-linear effects when following only the unbound solute, and the use of lipid membranes not fully hydrated when using solid-state NMR. Another significant challenge is the inherently low sensitivity of NMR, which often requires solute concentrations in the millimolar range. This can cause solubility issues in the aqueous phase and lead to high local concentration in the membrane, potentially perturbing its properties. Sensitivity improves with increasing NMR magnetic field strength and with the use of cryogenic probes, and significant advances are expected in the coming years for applying this technique to the characterization of solute–membrane interactions.

Another technique that has been occasionally used to evaluate solute–membrane interactions is electron paramagnetic resonance (EPR). Because most biologically relevant small molecules do not present paramagnetic properties, these studies are mostly limited to lipophilic electron spin probes [18,266,267,268,269,270,271] or solutes labeled with electron spin probes [272,273,274]. Indirect information on solute–membrane interactions has also been obtained from the effect of paramagnetic solutes on the NMR signal of membrane lipids [247,275] or solute effects on membrane properties reported by EPR probes [265,266,276,277,278,279]. However, these indirect studies are only qualitative or semi-quantitative and reflect association at membrane-perturbing conditions.

#### 3.1.3. Indirect Quantification of the Solute from Changes in the Properties of the Whole System and/or in the Membrane Properties

Due to the low concentration of solute expected in vivo (see Section 2.1) and in order to enable comparisons among different solutes, membrane compositions, and experimental conditions, it is generally preferable to characterize membrane affinity using very low solute concentrations, thereby avoiding membrane perturbation. However, most indirect methods rely on detecting changes in membrane properties and therefore typically require high local solute concentrations, often approaching membrane saturation.

This section presents the most common methods used to characterize solute–membrane partitioning that rely on changes in the properties of the membranes and/or the aqueous medium. The methods discussed range from isothermal titration calorimetry (ITC), which allows determination of affinity at both low and high solute concentrations, to techniques based on changes in membrane properties, such as electrostatic characteristics, liposome size, and solid–liquid phase transitions. Finally, methods based on changes in the properties of supported lipid membranes are briefly discussed.

##### Isothermal Titration Calorimetry (ITC)

In ITC, the property measured is the heat variation upon transfer of the solute from the aqueous media to the membrane, which depends on solute–solute, solvent–solvent, and solute-solvent interactions. In the more conventional approach, aliquots of a concentrated membrane solution are added to a solute solution, and the heat evolved after each addition is recorded. If the solvent in the membrane and solute solutions is perfectly matched and does not alter its properties in the concentration range evaluated (no heat of dilution), the heat evolved due to the addition of the membrane solution aliquot i, is proportional to the enthalpy variation of the association (${\Delta H}^{o}$) and to the variation in the number of moles of solute associated with the membrane due to the increase in the lipid concentration, ${∆}^{i}n_{\mathrm{SL}}$, Equation (29).

(29) $$q_{i}={\Delta H}^{o}\;\Delta^{i}n_{\mathrm{SL}}$$

The variation in the moles of solute associated with the membrane may be calculated from the dependence of $q_{i}$ with the lipid concentration, allowing calculation of the association affinity. Some methodological details may be consulted, for example, in references [97,185,280,281].

The sensitivity of the initial ITC instruments was in the range of μcal s^−1^ and therefore high concentrations of solute were needed, leading to very high local concentrations of solute in the membrane. In fact, most studies were dedicated to the study of membrane solubilization by high concentrations of amphiphilic solutes (e.g., [282]). However, modern ITC equipment has sensitivity in the range of ncals^−1^, allowing the use of much lower solute concentrations and thus the characterization of solute association to essentially unperturbed membranes [97,185]. It should be noted that membrane perturbation may be particularly problematic in experiments with the successive addition of membrane-enriched aliquots because the different titration steps are not independent and because higher local concentrations of solute are attained in the first titration points. Membrane perturbation is therefore higher in the beginning of the titration, with the perturbation propagating to all the titration curves.

The simpler model to describe the dependence of ${∆}^{i}n_{\mathrm{SL}}$ with the lipid concentration for small and uncharged solutes is considering a simple partition (Equation (1)) (e.g., [185]). If the solute is charged, electrostatic effects may need to be included in the model, allowing the characterization of the partition coefficient at the experimental conditions followed ($K_{P}$), and the intrinsic partition coefficient in the absence of electrostatic interactions ($K_{P}^{0}$) (Equation (14)) (e.g., [96,263]). If high local solute concentrations in the membrane are attained during the titration and/or the ionic strength of the aqueous media is low, the electrostatic potential at the membrane surface changes throughout the titration, and the data must be analyzed with the complete model, including electrostatic effects (details in Section 2.1 and analysis applications provided in references [97,98,185]). However, if the aqueous media contain high concentrations of ions (e.g., 0.15 M NaCl), if the solutes have a single charged group, and if solute local concentrations in the membrane are lower than 5 mol%, the membrane surface potential is small and maintained throughout the titration, allowing the use of the simple partition model to analyze the data and the calculation of the intrinsic partition coefficient using Equation (14) [97].

**Figure 8:**
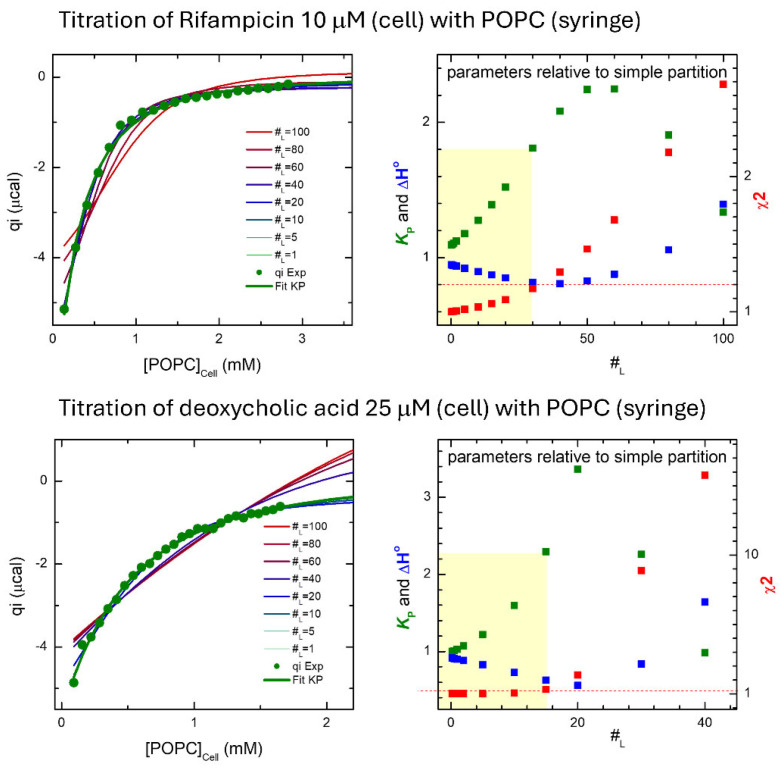
Dependence of the best fit and parameters recovered when the association of rifampicin (top figures) and deoxycholic acid (bottom plots) is analyzed with a model considering one set of independent binding sites, Equation (8). The plots on the left show the heat *per* peak obtained experimentally for the addition of 10 µL aliquots of POPC LUVs to the ligand in the ITC cell (●), and the best fit considering that ligand association with the membranes occurs by a simple partition (**—**) or by binding to independent binding sites in the membrane with ${\#}_{L}$ lipids *per* binding site, considering different values of ${\#}_{L}$ from 1 (—) to 100 (—). The plots on the right show the parameters obtained from the best fit for the distinct values of ${\#}_{L}$ relative to the parameters obtained from the best fit of a simple partition; $\Delta H^{o}$(■), and $K_{P}$ (■) calculated from $K_{b}$ and ${\#}_{L}$ using Equation (10). The relative quality of the best fit is also shown, χ2 (■). The horizontal red line marks a 20% increase in χ2, upper limit in a confidence interval of 80%, and the yellow boxes highlight the corresponding range of parameter values obtained.

The partition models discussed in Section 1 are not available in the analysis software provided by the ITC manufacturers, which is focused on ligand–protein binding. For that reason, some authors analyze the results assuming binding of the solute to well-defined binding sites in the membrane (e.g., [283]). Although this model does not adequately describe the association of small solutes with lipid membranes, the quality of the best fit obtained is usually good, providing estimates of the binding constant ($K_{b}$) and the size of the binding sites in the membrane (number of lipid molecules per binding site, ${\#}_{L}$), from which the partition coefficient may be calculated using Equation (10). A major concern when following this approach is that the estimates obtained for $K_{b}$ and ${\#}_{L}$ correspond to a set of values in a wide range of possible parameter combinations. This is illustrated in Figure 8 for the case of the association of the antibiotic rifampicin (data from reference [284]) and the surfactant deoxycholic acid (data from reference [119]) with POPC LUVs. If the number of binding sites available in the membrane is much larger than the number of solute molecules bound, the quality of the best fit is independent on ${\#}_{L}$, and only the ratio $K_{b}/{\#}_{L}$ may be obtained. The value of $K_{b}$ cannot, therefore, be used as a direct estimate of the solute–membrane affinity. However, this procedure allows the calculation of accurate estimates for $K_{P}$, using Equation (10), and $\Delta H^{o}$. The yellow box in the right plots of Figure 8 highlights the range of ${\#}_{L}$ values that lead to a best fit quality similar to that obtained considering the simple partition model ($\chi^{2}\leq 1.2\;\chi_{partition}^{2}$, corresponding to the upper limits of an 80% confidence interval). For the case of titration of 10 µM rifampicin, this interval corresponds to ${\#}_{L}$ up to 30, while the maximum number of lipids per binding site is decreased to 15 in the case of titration of 25 µM deoxycholic acid. The different intervals reflect the distinct maximum local concentrations achieved, with 90 lipid molecules per solute bound in the case of rifampicin and 50 lipid molecules per solute bound in the case of deoxycholic acid. At the limit of the 80% confidence interval, the value of $K_{P}$ is overestimated by approximately two-fold and $\Delta H^{o}$ is underestimated by about 50%. The deviations from the true parameters are comparable to other experimental and model uncertainties such as the fraction of lipid accessible due to liposome multilamellarity and/or slow solute translocation (see Section 1 for details). Therefore, provided that the solute–membrane affinity is reported as ${n\;K}_{b}$, n being the number of binding sites *per* lipid, the best fit using the *one set of sites model* available in the analysis software provided by the ITC manufacturer is a reasonable approach to describe the association of small molecules with lipid membranes. Given the comparable size of the solutes and lipids, it is more meaningful to define the binding sites in terms of the number of lipid molecules in each binding site (${\#}_{L}$), and not as the number of binding sites per lipid (n), with ${\#}_{L}=1/n$. In any case, this parameter is usually not very informative given that the best fits with similar quality are obtained from distinct sets of parameter values (n and $K_{b}$). The partition coefficient ($K_{P}$) may, however, be calculated from $n\;K_{b}/{\bar{V}}_{L}$ or the equivalent $K_{b}/\left({{\#}_{L}\bar{V}}_{L}\right)$, Equation (10).

The Langmuir adsorption model (Langmuir isotherm) has also been used to describe solute association with lipid membranes (e.g., [285]). This model is in fact equivalent to the *one set of sites* model with the size of the binding sites described as a surface area.

In addition to providing acceptable estimates of the partition coefficients and association enthalpy variations for small solutes, the explicit consideration of binding sites in the membrane may in fact be the most adequate model for the interaction of some specific solutes and membranes, namely in the case of large solutes with multiple charged groups and membranes with high surface charge density (e.g., [66]). It has nevertheless been shown that these systems may usually still be well described by partition models provided that the contributions from electrostatic interactions are included (e.g., [286]).

Complementing the most usual approach of titrating the solute with increasing concentrations of lipid, some applications are based on the titration of the membrane with increasing concentrations of solute. This approach has mostly been used with only a few titration steps to avoid large variations in the solute concentration with the goal of obtaining the interaction enthalpy and the assumption of full solute binding (e.g., [287]). When a full titration is performed, the heat evolved usually decreases with the increase in the solute concentration, and this has been interpreted as saturation of independent binding sites in the membrane and analyzed with the *one set of sites* model (Equations (7)–(8)) (e.g., [66,283,288]). However, the assumption of independent binding sites in the membrane is not usually valid, and while for solute titration with lipid this analysis may provide reasonable estimates of the association affinity, the decrease in the amount of solute that associates with the membrane at high solute concentrations is in fact due to changes in the membrane properties.

**Figure 9:**
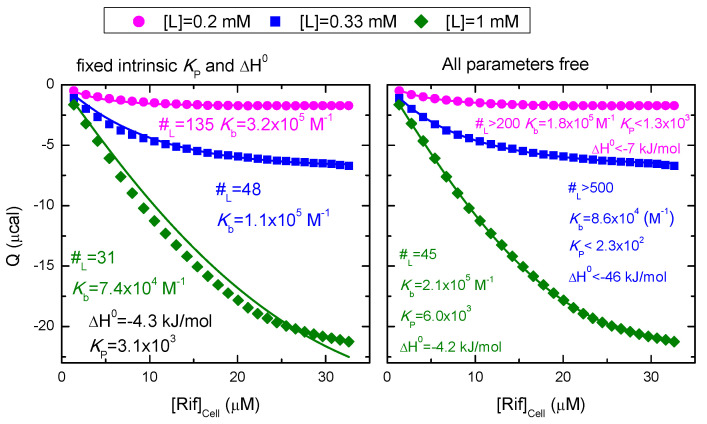
The binding affinity obtained from saturation curves in ITC. The symbols correspond to the cumulative heat variation when 10 µL aliquots of rifampicin 200 µM are added to a POPC solution in the ITC cell ($V_{Cell}$ =1436 µL) containing a lipid concentration of 0.2 mM (●), 0.33 mM (■), or 1 mM (♦). The lines correspond to the best fit for binding to one set of independent sites (Equation (8)) with the parameters shown in the figure with the same color used for the data points. The binding affinity and enthalpy variation were fixed at the parameter values for unperturbed membranes (obtained from titrations varying the lipid concentration), while in the right plot they were adjusted to obtain the best fit.

A combination of these two approaches (lipid addition to solute in the cell or solute addition to lipid in the cell) has been proposed to characterize the membrane association of very low affinity solutes [284], and the data obtained from both sets of titrations may provide a critical evaluation of the use of binding sites to characterize solute–membrane affinity. In that approach, several independent titrations are performed by adding the solute to distinct lipid concentrations in the cell. In each titration, increasing solute concentrations are achieved, providing information regarding membrane saturation from the dependence of the heat evolved ($q_{i}$) with solute concentration. The association to unperturbed membranes may, however, be obtained from the dependence of $q_{i}$ of the first solute addition for independent titrations with distinct lipid concentrations. This approach has two advantages. First, the lipid concentrations that may be attained in the cell are much higher than when following the usual approach of adding the lipid membranes to the solute in the cell. Second, all titration data points are independent and report the interaction with unperturbed membranes. A drawback is, however, the need to perform several independent titrations to obtain a single estimate of the partition coefficient. To assess the adequacy of obtaining solute affinity from the variation of the heat with the solute concentration (that is, from the saturation curve in each titration curve), data obtained in reference [284] for the interaction of rifampicin with some representative concentrations of POPC were reanalyzed and are shown in Figure 9.

The left plot shows the best fit of a model considering binding sites in the POPC membrane (Equation (8)), but fixing the partition coefficient and enthalpy variation at the intrinsic parameter values obtained from the dependence of $q_{i}$ with the lipid concentration ($K_{P}$ = 3.1 × 10^3^ and ${\Delta H}^{o}=-$4.3 kJ/mol). A reasonable fit is obtained, especially for low lipid concentrations, providing an estimate of the number of lipids per binding site (${\#}_{L}$) and binding constant ($K_{b}$, obtained from Equation (10)). The right plot shows the best fit obtained when all parameters are allowed to adjust freely. As expected, the quality of the best fit improves, leading to excellent fits. However, the binding constant ($K_{b}$) is the only parameter that is reasonably consistent for the different lipid concentrations, whereas the estimates of $K_{P}$ and ${\Delta H}^{o}$ are significantly different from those obtained for the interaction with unperturbed membranes and vary widely for the different lipid concentrations. Also, in most cases, an accurate estimate of the parameters could not be obtained, only an upper or lower limit.

The analysis shown in Figure 9 shows that the solute saturation curves obtained by titrating the membrane with increasing solute concentrations do not lead to adequate parameters when analyzed with the assumption of well-defined binding sites in the membrane. Therefore, although this model may be justified in the case of the association of large multivalent ions with membranes of opposite charge, where binding is mostly due to electrostatic interactions [66,289], the results may still be described by a simple partition, regardless of the fact that the contribution from electrostatic interactions is included [289,290]. Moreover, distinct association parameters are obtained when following both approaches (addition of lipid to ligand or vice versa) [289], and more complex models may be required to adequately describe the association of solutes with membranes, including solute–membrane and solute–solute interactions (e.g., [215,291]). The parameters obtained from solute saturation curves should therefore be interpreted with caution, at least for the interaction of small ligands when followed by ITC.

The application of ITC to characterize solute–membrane interactions goes far beyond obtaining the interaction affinity. A major advantage is the full characterization of the variation in the thermodynamic parameters of the system associated with the transfer of the solute from the aqueous media to the membrane ($\Delta G^{o}$, $\Delta H^{o}$, and $\Delta S^{o}$), providing important insight into the type of interactions established and/or changes in solute/membrane properties [106,292,293]. For this goal, the definition of the reporter affinity parameter is of major importance because this determines the magnitude of $\Delta G^{o}$, from which $\Delta S^{o}$ is calculated (Equation (30)).

(30) $$\Delta G^{o}=-RT\;ln\;K=\Delta H^{o}-T\Delta S^{o}$$

From studies of hydrocarbon partition between water and organic solvents, evidence has been obtained for a higher adequacy of partition coefficients defined as volume fraction corrected for the distinct volumes of the species involved to account for variations in the mixing entropy (Flory–Huggins theory) [294,295]. However, controversy remains regarding the most appropriate formalism for partitioning lipid membranes [292,296]. The impact of the definition of the partition coefficient on $\Delta G^{o}$—and correspondingly on $\Delta S^{o}$—is given in Equation (31) for $K_{P}^{n/m}$ and $K_{P}$.

(31) $$\Delta G^{o}\left(K_{P}^{n/m}\right)=\Delta G^{o}\left(K_{P}\right)-RT\ln\frac{\rho_{W}}{\rho_{M}}≅\Delta G^{o}\left(K_{P}\right)$$

(32) $$\Delta G^{o}\left(K_{P}^{n/n}\right)=\Delta G^{o}\left(K_{P}\right)-RT\ln\left({\bar{V}}_{L}\left[W\right]\right)≅\Delta G^{o}\left(K_{P}\right)-10kJ/mol$$

In the case of a solute with $K_{P}=1\times 10^{3}$ (leading to $\Delta G^{o}=-18\;kJ/mol$), a value of $\Delta H^{o}=-20\;kJ/mol$ would predict $T\Delta S^{o}=-2\;kJ/mol$. If the same membrane affinity is expressed as a molar fraction, $K_{P}^{n/n}=4\times 10^{4}$ (leading to $\Delta G^{o}=-28\;kJ/mol$), the same $\Delta H^{o}$ would predict $T\Delta S^{o}=8\;kJ/mol$. Thus, although in both cases it is concluded that the solute–membrane association is driven by enthalpy, the use of $K_{P}$ predicts a small decrease in the system entropy, while a significant increase would be predicted if the partition coefficient is reported as $K_{P}^{n/n}$. The estimated contribution of entropy variation to the solute–membrane affinity will depend on $\Delta G^{o}$ and $\Delta H^{o}$, with $T\Delta S^{o}$ being always 10 kJ/mol higher (more positive or less negative) if $\Delta G^{o}$ is calculated from $K_{P}^{n/n}$ instead of $K_{P}$.

Another important application of ITC is in the characterization of changes in ionization due to solute–membrane association. The enthalpy variation directly measured in an ITC titration reflects changes in the system upon transfer of the solute from the aqueous phase to the membrane. It includes changes in the interactions established by the solute with both solvents, as well as other concomitant processes involving heat variations. These include solvent–solvent interactions, which contribute directly to the solutes’ partition affinity, but also other indirect changes in the system components. An important example is the case of changes in the ionization of solute or membrane lipids, which is accompanied by H^+^ release or capture by the pH buffer in the aqueous media. Depending on the enthalpy variation associated with H^+^ dissociation from the buffer ($\Delta H_{buffer}^{o,diss}$), the enthalpy variation directly obtained may differ significantly from that associated with solute transfer between the aqueous media and the lipid membrane ($\Delta H_{P}^{o}$), the difference being related to the number of H^+^ captured by the solute/lipid (released by the buffer) due to solute transfer from the aqueous media to the membrane ($\Delta n_{H^{+}}$, Equation (33)).

(33) $$\Delta H^{o}=\Delta H_{P}^{o}+\Delta n_{H^{+}}\Delta H_{buffer}^{o,diss}$$

The dependence of $\Delta H^{o}$ on the pH buffer properties is both an inconvenience and an opportunity. On the one hand, to obtain a full characterization of the thermodynamic parameters associated with solute partition from a single titration, it is mandatory to use buffers with small $\Delta H_{buffer}^{o,diss}$ such as inorganic phosphate [297,298]. On the other hand, by performing distinct titrations using buffers with distinct ionization enthalpies, it is possible to obtain $\Delta n_{H^{+}}$. When the membrane is formed from non-ionizable lipids and/or phosphatidylcholines only, the H^+^ transferred may be directly attributed to the solute and allow characterizing changes in solute ionization upon association with the membrane. This approach has been followed to obtain the charge of several drugs and natural biomolecules when associated with lipid membranes, providing important insights regarding the mechanisms of their bioavailability and bioactivity [3,119,213,286,287,299,300].

ITC can also provide important information regarding the kinetics of solute–membrane interactions. The uptake and release protocol proposed by Heerklotz and co-workers provides semi-quantitative information on the rate of solute translocation between the membrane outer and inner leaflets through the combination of two types of titrations [301]. The uptake titration follows the usual ITC procedure, with liposomes added to the solute in the ITC cell, while in the release titration liposomes previously equilibrated with the solute are added to the aqueous buffer, thus reporting on the heats evolved due to the release of solute from the liposomes. The global analysis of both titrations provides the association affinity, the enthalpy variation, and the fraction of lipid accessible to the solute during each titration step. This information is related to the rate of solute equilibration between the two membrane leaflets and also to possible multilamellarity on the liposomes. Additional limitations that prevent the use of this approach to quantitatively obtain the rate of solute translocation include the low rate of titrant addition in the ITC experiment (typically 0.5 µL/s, preventing the characterization of fast translocation processes) and the propagation of the heat evolved in the first titration steps throughout the whole titration in the case of slow kinetics. Nevertheless, this approach allows classification in terms of slow, intermediate, or fast permeating solutes [97,119,213,300,302,303,304]. Transformations that occur on the time scale of a few minutes may also be characterized directly through the time dependence of the heat evolved after each titration step. This approach has been used in the case of the interaction of chlorpromazine with liposomes of distinct lipid compositions [97]. After the addition of the liposome aliquot, a large heat variation was observed that reflected the fast association of the solute with the membrane. The full equilibration of the solute with the membrane (that is, the return to baseline) involved, however, an additional slow step that occurred on the time scale of several minutes and could therefore be quantitatively characterized. After exclusion of contributions from membrane multilamellarity, this slow step was attributed to solute translocation between the outer and inner membrane leaflets. Contributions from other slow processes involved in the full equilibration of charged solutes across lipid membranes cannot, however, be discarded [305,306].

As a final remark, despite its potentially high utility, the characterization of solute–membrane interactions by ITC is not always straightforward. This difficulty arises primarily from the small enthalpy changes expected for associations that are largely driven by the hydrophobic effect. Although the establishment of electrostatic interactions between the solute and lipid polar groups can produce measurable heat changes, these variations are typically limited to at most a few tens of kJ/mol. Moreover, such interactions are often of low to moderate affinity, resulting in the distribution of the total heat change over many titration steps and, consequently, a further reduction in the heat *per* injection. To accurately characterize the interactions of small molecules with lipid membranes, high-purity lipids, perfectly matched solvents, and ITC instrumentation in optimal condition are required. A standard operating procedure has recently been published that provides important recommendations, including minimization of solvent mismatch and detailed equipment cleaning and calibration procedures [307].

##### Methods Based on Changes in the Membrane Electrostatic Properties

The membrane association of charged solutes may be characterized through variations of the membrane surface charge density ($\sigma_{0}$), and corresponding surface potential ($\psi_{0}$) at increasing solute concentrations. This property may be obtained from the liposome electrophoretic mobility ($\bar{u}$) or zeta potential (ζ) [3,45,161,200,213,234,308,309,310,311], knowing the ionic strength and viscosity of the aqueous media and the distance between the membrane surface and the liposome slipping plane ($d_{\zeta }$) (Equation (34)) [234,309]:

(34) $$\zeta =\frac{2kT}{e}ln\frac{1+\alpha exp\left(-\kappa d_{\zeta }\right)}{1-\alpha exp\left(-\kappa d_{\zeta }\right)};\alpha =\frac{exp\left(e\psi_{0}/2kT\right)-1}{exp\left(e\psi_{0}/2kT\right)+1}$$

where κ is the inverse of the Debye length ($\lambda_{D}$), given by Equation (18)).

Evidence for $d_{\zeta }$ = 2 Å has been obtained for the case of membranes from pure phosphatidylserine and mixtures with phosphatidylcholine in aqueous media containing monovalent salts [100,234], and this value is commonly used to calculate the surface potential from the measured zeta potential. The membrane electrostatic properties can also be obtained using membrane-bound fluorescent sensors (e.g., fluorescein phosphatidylethanolamine or di-8-ANEPPS to report on the membrane surface and dipole potential, respectively), and this has been used to characterize the membrane affinity of charged peptides [312].

Because the affinity is obtained from changes in the membrane electrostatic properties, to obtain the intrinsic affinity it is always necessary to correct for the effects of the surface potential on the association affinity. This is usually done using the Gouy–Chapman theory (Section 2.1).

An important limitation of these methods is the assumption of additivity for the contribution from the solute and lipids and that their electrostatic properties are independent of the local concentrations in the membrane. Another concern is that they require knowing the average charge of the solute when associated with the membrane, a parameter that is not always well known due to changes in the solute ionization equilibria when associated with the membrane. On the other hand, when the membrane affinity is known from independent experiments performed at the same conditions, these methods allow the characterization of the solute average charge when associated with the membrane (e.g., [3]).

##### Methods Based on Changes in the Liposome Size

Thermophoresis is a property based on the directed motion of molecules or supramolecular entities induced by temperature gradients, which, depending on the molecular properties and temperature, leads to a depletion (thermophobicity) or accumulation (thermophilicity) in the region with a higher temperature [313]. The concentration gradient depends on the diffusion coefficient which allows the characterization of the size of the diffusing entity [314] and in turn may be used to characterize molecular interactions. This methodology is increasingly being used to evaluate ligand-protein binding affinities [315,316,317], and has also been applied to characterize the membrane-binding affinity of proteins and peptides [318,319] and other bioactive molecules [320,321,322]. To eliminate contributions from solvent flow, the sample is placed in thin capillaries of 100 µm diameter. This methodology requirement is in fact one of the major advantages of this approach, requiring the use of very small amounts of sample. The concentration variations in the heated region of the sample are usually visualized by fluorescence, the method thus requiring the use of fluorescent binding agents, either resulting from the presence of intrinsic fluorescent moieties or after their modification with fluorescent probes. In a typical experiment, the concentration of binding agent is maintained constant, and the dependence of the fluorescence intensity on the ligand concentration is followed over time during the establishment of the temperature gradient. The saturation fraction of the binding agent with the ligand (f) is then calculated from the variation in the fluorescence intensity at a given ligand concentration (${\Delta c}_{i}$), which depends on the variation observed in the absence of ligand (${\Delta c}_{unbound}$) and at full saturation of the binding agent (${\Delta c}_{bound}$) (Equation (35)). The binding affinity may then be calculated using the most adequate binding formalism which may include a set of several distinct binding sites with or without binding cooperativity [67,323].

(35) $${\Delta c}_{i}=f{\Delta c}_{bound}+\left(1-f\right){\Delta c}_{unbound}$$

When applied to the characterization of ligand–membrane binding, this methodology suffers from the same limitations as previously indicated for all methods that rely on membrane saturation with the ligand and thus do not provide the parameters for association to unperturbed membranes.

In the case of large solutes and/or at high local solute concentrations, the association of the solute with the membrane increases the liposome size, and this may be followed directly by dynamic light scattering (DLS). As indicated above, if the liposome size is followed at increasing solute concentrations, the results provide only information regarding the membrane saturation with the solute. However, data can also be obtained for a fixed solute concentration at increasing liposome amounts. In this case, the liposome size is larger at low lipid concentrations (due to the high local concentration of solute) and tends towards the size of pure liposomes as the solute–lipid ratio decreases. This approach has been followed to characterize the association of a lipidic a-amino acid with POPC liposomes, leading to a partition coefficient comparable to that obtained by ITC [213]. In this work, the variation in the liposome size was however very small (less than 2 nm radius for liposomes with a radius of 55 nm) and required the use of high local concentrations of solute (above 10 mol%), highlighting the low sensitivity of the method for small solutes. Although with limited application to characterize quantitatively the solute–membrane affinity, DLS is an important complementary technique, providing information regarding the effects of the solutes on membrane properties (e.g., [47,324,325,326]).

##### Methods Based on Changes in the Lipid Phase Transitions

The temperature of the phase transition of lipid membranes depends on the presence of foreign molecules [327], similarly to solute effects on the freezing temperature depression in homogeneous media, and this may be used to follow the association of solutes with membranes. A major advantage of this method is that it may be used with any solute, without requiring any specific solute property such as charge or fluorescence. The changes in the lipid phase transition may be followed by differential scanning calorimetry (e.g., [261,328,329,330,331]) but also by turbidimetry due to the increase in the refractive index of membranes in the gel phase (e.g., [332,333,334,335]), or by changes in the fluorescence properties of membrane-embedded probes such as in the anisotropy of diphenylhexatriene (e.g., [199,200,336,337]).

The formalism to analyze the decrease in the melting temperature is based on the van’t Hoff equation for freezing-point depression [338]. If the solute associates only with the fluid membrane, the decrease in the membrane phase transition temperature is given by Equation (36):

(36) $$\Delta T_{m}=-\frac{R{\left(T_{m,0}\right)}^{2}}{\Delta H_{m,0}^{o}}\frac{K_{P}^{n/n}}{\left[W\right]+K_{P}^{n/n}\left[L\right]}\left[S_{W}\right]$$

where $T_{m,0}$ and $\Delta H_{m,0}^{o}$ are the melting temperature and the melting enthalpy of the lipid in the absence of solute, $K_{P}^{n/n}$ is the partition coefficient of the solute defined in terms of mole fraction (Equation (3)), and $\left[W\right]$, $\left[L\right]$, and $\left[S_{W}\right]$ are, respectively, the concentration of water, of lipid in the fluid phase (half the total lipid at $T=T_{m}$), and the concentration of solute in the bulk aqueous phase, all defined as molar fraction.

If the fraction of solute associated with the membrane is negligible—obtained at low lipid concentrations or for solutes with weak membrane-affinity—Equation (36) may be simplified to Equation (37) and a linear dependence is observed between $\Delta T_{m}$ and the total solute concentration $\left[S\right]$.

(37) $$\Delta T_{m}=-\frac{R{\left(T_{m,0}\right)}^{2}}{\Delta H_{m,0}^{o}}K_{P}^{n/n}\left[S\right]$$

The assumption that solutes dissolve only in the melted (fluid) phase does not always hold for lipid membranes. Some lipids, such as phosphatidylcholines—the main lipids in biomembranes—can form an intermediate phase between the gel and fluid states, which can incorporate substantial amounts of solute [339,340]. When the partition coefficient is estimated from the melting temperature depression using Equation (36), what is being reported is the difference between solute partitioning into the membrane above and below the melting temperature. If solute binding to the phase below the melting point is significant, the method underestimates the true partition coefficient between the aqueous phase and the fluid membrane [341].

The general equation for the decrease in the melting temperature is given in Equation (38) [338].

(38) $$\Delta T=-\frac{R{\left(T_{m,0}\right)}^{2}}{\Delta H_{m,0}^{o}}\;\frac{K_{1}^{n/n}-K_{2}^{n/n}}{\left[W\right]+\left\{\alpha K_{1}^{n/n}+\left(1-\alpha \right)K_{2}^{n/n}\right\}\left[L\right]}\;\left[S\right]$$

where $K_{i}^{n/n}$ is the partition coefficient between the aqueous media and membrane phase just above $\left(i=1\right)$, and below $\left(i=2\right)$ the melting temperature, and α is the mole fraction of lipid in the melted phase at the temperature evaluated (α = 0.5 at the midpoint of the transition).

Using Equation (38) to analyze the dependence of the melting temperature on the solute concentration at different concentrations of lipid, the partition coefficients of several drugs between the aqueous media and DPPC membranes in the fluid and in the intermediate phase (the rippled phase) were obtained [338]. The ratio between the two partition coefficients was equal to two or less, indicating that the partition coefficients for the fluid phase are underestimated by 50% or more if the data is analyzed with the simplified Equation (36). The partition into DPPC above and below the main phase transition (from rippled to gel) was characterized by Kawamura *et al.* for a homologous series of benzyl alkanols from phenol to 8-phenyl-1-octanol [342]. It was found that the relative partition into the less fluid rippled phase increased with the length of the alcohol (being negligible for n≤4 and increasing up to 30% for n=8). The authors also analyzed the effect of the solutes on the temperature of the pre-transition (from gel to rippled) and found negligible association of the solutes with the more ordered gel phase. These studies show that variations in the membrane main phase transition may be used to report quantitatively the association of solutes with membranes where the main transition is from gel to fluid phases and when the solute shape is much different from that of the membrane lipids. However, it must be used with caution when the gel-to-fluid transition involves phases with intermediate properties and/or when the solutes have lipid-like structural properties. For this reason, the measurement of changes in the transition temperature is a methodology commonly used, but only for a qualitative assessment of solute–membrane association (e.g., [90,134,264,343,344,345,346,347,348]).

##### Methods Based on Interactions with Solid-Adsorbed Membranes

Nanoplasmonic sensors with applications in lipid membrane studies include surface plasmon resonance (SPR) and quartz crystal microbalance (QCM). The details of these methodologies have been reviewed recently, including the principles involved and most common applications [349]. A major advantage is that the solute dissociation rate constant from the membrane may be easily characterized, providing information on the interaction kinetics.

Common limitations of these methodologies include the use of membranes attached to solid supports, with possible effects on membrane structural and dynamic properties, and the fact that titrations are performed at increasing concentrations of solutes, where the solute–membrane affinity is obtained from membrane saturation curves. The use of immobilized membranes greatly simplifies the experimental approach and allows the characterization of a large number of solutes with the same membrane preparation. Therefore, although most commonly used in the characterization of solute-macromolecule interactions, these methodologies have also been used to characterize solute–membrane interactions [215,350,351,352,353,354,355,356,357,358,359,360].

The formalism to obtain partition coefficients from the variations in the SPR signal at increasing solute concentrations has been recently derived [351] and is given by Equation (39), where ${RU}_{L}$ and ${RU}_{S}$ correspond to the SPR signal in the absence and presence of solute, respectively, $M_{L}$ and $M_{S}$ are the molar masses of lipid and solute, and σ is the lipid-to-solute ratio at saturation (equivalent to the number of lipids *per* binding site, ${\#}_{L}$).

(39) $$\frac{{RU}_{S}}{{RU}_{L}}=\;\frac{\bar{V_{L}}K_{P}\frac{M_{S}}{M_{L}}\left[S_{T}\right]}{1+\sigma \bar{V_{L}}K_{P}\left[S_{T}\right]}\;$$

The assumptions in the derivation of this equation are as follows: (i) a linear dependence of the SPR signal on the mass of the membrane in the sensor (including the membrane and the membrane-associated solute); (ii) that the fraction of solute associated with the membrane is negligible ($\left[S_{W}\right]≅\;\left[S_{T}\right]$); and (iii) that the solute affinity for the membrane is maintained ($K_{P}$ independent on $\left[S_{M}\right]$) with the volume of membrane available for solute partition decreasing with the amount of solute associated with the membrane and being given by $\sigma \bar{V_{L}}\left[L_{T}\right]$. While assumptions (i) and (ii) are generally considered in SPR, assumption (iii) assumes that the solute interacts with well-defined binding sites in the membrane and its validity may depend on the solute and membrane. This approach has been followed to characterize the interaction of a protein ($M_{W}$ = 16.44 kDa), and two peptides ($M_{W}$ = 4.492 and 0.337 kDa) with POPC membranes, leading to σ values of 9.3, 50, and 69, respectively [351]. The observation that the ratio of lipid to solute at saturation (that is, the size of the binding site) is inversely related to the solute size points towards some possible pitfalls in the application of this approach to obtain the solute–membrane affinity parameters. Nevertheless, the partition coefficients obtained are in reasonable agreement with those obtained independently by changes in the fluorescence quantum yield of the solute, highlighting the potential of this methodology at least to obtain relative membrane affinities, with the advantage of providing information on the interaction kinetics as well [361,362,363,364,365,366,367].

### 3.2. Computational Approaches

#### 3.2.1. Molecular Dynamics Simulations

Computational tools are becoming a standard methodology to evaluate solute–membrane interactions, with molecular dynamics (MD) simulations under NPT conditions being of particular relevance (e.g., [130,368,369,370]). Local partition coefficients, $K\left(z\right)$, are usually calculated from the excess free energy at the z coordinate relative to a reference position, $\Delta \Delta G^{o}\left(z\right)$ (Equation (40)).

(40) $$K\left(z\right)=e^{\frac{-∆∆G^{o}\left(z\right)}{RT}}$$

The solute–membrane affinity parameter in Equation (40) was intentionally undefined. It is, however, necessary to define whether it is $K_{b}$, or any specific partition coefficient, namely $K_{P}$ or $K_{P}^{n/n}$, because their quantitative relation with $\Delta G^{o}$ is different and would lead to the prediction of different solute–membrane affinities (Equation (32)). To select between the two reporters for solute–membrane affinity, it is necessary to understand how $\Delta G^{o}$ is obtained. In unrestrained simulations, what is characterized directly is the time that the solute stays associated with the membrane or in the water. The fractional time spent in each position along the z coordinate directly gives the probability density function by calculating a histogram with a discrete number of bins of equal width along the z-coordinate (that contains the membrane and water media). Since the bins have equal widths and xy areas, the local solute densities obtained for each bin can be treated as relative local concentrations in units of moles *per* unit volume. Hence, the partition coefficient obtained directly from the relative time spent at each z coordinate is defined as $K_{P}$ (Equation (1)).

The relation is not so obvious when the free energy profile is obtained from enhanced sampling simulations such as umbrella sampling, where the probability density is obtained under the application of a force [371]. Because $\Delta G^{o}$ is related to the partition coefficient (Equation (40)), the definition of $K\left(z\right)$ has an impact on $\Delta G^{o}$, and vice versa (see discussion in the Isothermal Titration Calorimetry (ITC) section, Equations (31) and (32)). So, before this issue is clarified, the corresponding uncertainty for the predicted solute–membrane affinities obtained from biased MD simulations must be included in the comparison with experimentally measured affinities. For simplicity, in the following analysis, it will be assumed that the solute–membrane affinity obtained from MD simulations (unrestrained or biased) is defined as $K_{P}$.

The local partition coefficients obtained from Equation (40) are required to calculate the permeability coefficients from the aqueous media on both sides through the membrane, according to the inhomogeneous solubility-diffusion mechanism [372]. The local partition coefficients cannot, however, be directly compared with those obtained experimentally, which report on the solute association with the whole membrane.

Different approaches have been proposed to calculate the overall partition coefficient (K, which following the discussion above, is assumed to correspond to $K_{P}$) from the free energy profile obtained in MD simulations. The simpler approach is to consider the partition between the aqueous media and the equilibrium position in the membrane ($z_{Eq}$), corresponding to the minimum in the free energy profile [373], Equation (41).

(41) $$K_{P}=e^{-\frac{\Delta G^{o}\left(W\to z_{Eq}\right)}{RT}}=e^{-\frac{\left(\Delta G^{o}\left(z_{Eq}\right)-\Delta G^{o}\left(z_{W}\right)\right)}{RT}}$$

This procedure has been successfully applied to obtain partition coefficients between two homogeneous solvents [6], where the free energy profile is mostly invariant within each phase. This is not, however, the case for lipid membranes, with $\Delta G^{o}\left(z\right)$ varying significantly with z (the transversal position across the lipid bilayer). The partition coefficient calculated from Equation (41) is related to the maximal membrane affinity, to a specific location in the membrane and, as expected, usually leads to an overestimation of the partition coefficients. Moreover, deviations will depend on the width of the equilibrium position [6,374,375], introducing uncertainty even in semi-quantitative analysis, such as in the ordering of distinct solutes. Those limitations have been pointed out since the first use of this approach [373] but are often overlooked [376].

A more realistic comparison between the partition coefficient obtained from the free energy profile and that obtained experimentally is obtained when the whole membrane is considered. This has first been done by MacCallum and Tieleman [377] for the distribution of hexane between water and a DOPC bilayer, Equation (42).

(42) $$\begin{array}{c} K_{P}=\frac{1}{5}\int_{-2.5}^{2.5}K\left(z\right)\;dz=\frac{1}{5}\int_{-2.5}^{2.5}e^{-\frac{\Delta \Delta G^{o}\left(z\right)}{RT}}dz\\ \Delta \Delta G^{o}\left(z\right)=\Delta G^{o}\left(z\right)-\Delta G^{o}\left(W\right) \end{array}$$

where *z* = 0 was defined for the nonpolar center of the membrane, and the limits of the integration correspond to the extreme coordinates of hexane distribution found experimentally (−2.5 nm < *z* < 2.5 nm) [378]. The integral is divided by the width considered for the bilayer (5 nm) to make the partition coefficient independent of the size of the system considered. This is the method that is being followed in most publications. However, the limits of the integration and the normalization factor have been modified by several authors. In most publications, the integration is only performed for one membrane leaflet, justified by the symmetry of the membrane with respect to both leaflets [214,375,379]. Regardless of the integration being for the whole bilayer or for one leaflet only—leading to equivalent results in the case of symmetric membranes—some authors use different integration limits. In some cases, this is done to include thicker membranes, with the integration being extended until a flat free energy profile in the aqueous media is obtained [214]. However, in some publications, the choice of the integration limits seems rather arbitrary [375,379], and surprisingly, at first, the normalization factor does not always correspond to the width of the integration [375].

Piasentin et al. [380] proposed an alternative equation to calculate the partition coefficient from the free energy profile (Equation (43)), where the simulation box is divided into several layers parallel to the membrane surface, $n_{W}$ corresponding to layers containing water, and $n_{L}$ to layers containing the lipid membrane. The distinction between both media is done on the basis of the solute-free energy profile, with the cut-off set at $\left\lceil ∆G^{o}\right\rceil$ > 0.2 kJ/mol.

(43) $$K_{P}=\frac{n_{W}\sum_{i=0}^{n_{L}}e^{-\frac{∆G\left(z_{i}\right)}{RT}}}{n_{L}\sum_{j=0}^{n_{W}}e^{-\frac{∆G\left(z_{j}\right)}{RT}}}$$

The box layers in Equation (43) containing bulk water do not, in practice, contribute to the calculated $K_{P}$ because $∆G^{o}≅0$ and $n_{W}/\sum_{j=0}^{n_{W}}e^{-≅0}≅\;$1. Thus, this equation is essentially equivalent to Equation (42) with the integration performed up to $\left\lceil ∆G\right\rceil$ > 0.2 kJ/mol and a pre-exponential factor corresponding to the width of the integration.

**Figure 10:**
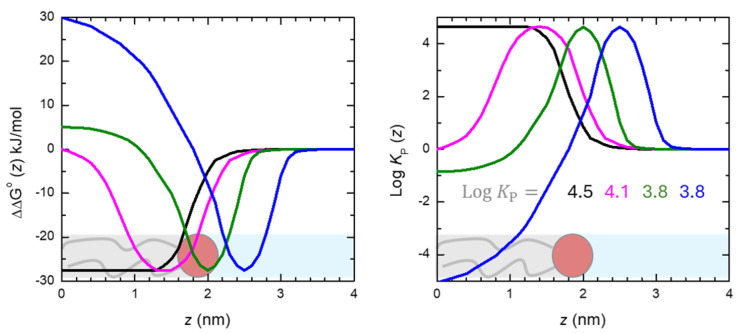
Hypothetical free energy profiles ($\Delta \Delta G^{o}\left(z\right)$, left plot) and logarithm of the corresponding local partition coefficients ($\log K_{P}\left(z\right)$, **right plot**) calculated from Equation (40) for the case of a very hydrophobic solute (**—**), a solute with intermediate hydrophobicity and a broad distribution in the membrane (**—**), and two more polar solutes with well-defined locations in the region of the lipid headgroups (**—**) or at the membrane/water interface (**—**). The location of the membrane and water are schematically represented by the lipid and the light blue box. The maximum value of $\log K_{P}$, calculated from Equation (41), is equal to 4.6 for all solutes, while the overall $\log K_{P}$ calculated from Equation (46) depends on the width of the free energy well and varies from 4.5 to 3.8. The parameters considered in Equation (46) were h = 2 nm for the pre-exponential factor and integration from z = 0 to z = 3 except for the solute adsorbed at the membrane/water interface, where the upper limit was z = 4. Schematic scaled representations of phospholipid molecules (with the head groups and acyl chains depicted as red circles and gray lines, respectively) are shown for the sake of illustration.

The free energy profile and corresponding local partition coefficient of a set of hypothetical solutes are shown in Figure 10 to illustrate the effect of the different methods discussed above for the determination of $K_{P}$. To facilitate the comparison, the minimum of $\Delta \Delta G^{o}\left(z\right)$ is the same for all solutes; only the width of the free energy well and the coordinate of the minimum are changed. The curves shown in black represent a small and very hydrophobic solute whose center of mass (COM) may be located at any of the z coordinates of the lipid nonpolar acyl chains. On the other extreme, the curves in blue represent a polar solute that interacts only with the membrane polar region with its COM located at the membrane/water interface. The additional two solutes (magenta and green curves) represent solutes with intermediate polarity where the width and location of the $\Delta \Delta G^{o}\left(z\right)$ minimum is varied.

Because the minimum of $\Delta \Delta G^{o}\left(z\right)$ is the same for all solutes considered, the value obtained for $K_{P}$ when using Equation (41) is also the same, being equal to 4.4 × 10^4^ (Log $K_{P}$ = 4.6). However, a higher membrane affinity would be expected for the solutes with a broader distribution in the membrane, and this is captured by the overall partition coefficients calculated from the integration of $K_{P}\left(z\right)$. This calls attention to the limitations of using Equation (41) for the calculation of the solute–membrane affinity. The exact value obtained for $K_{P}$ depends on the integration limits and pre-exponential factor. While the integration limits should include all the z coordinates with $\Delta \Delta G^{o}\left(z\right)\neq 0$, the region where the solute free energy is affected by the presence of the lipid membrane, the choice of the pre-exponential factor deserves some discussion.

If the goal is to obtain the average concentration of solute when associated with the membrane, then a pre-exponential factor equal to the width of the integration interval is the most adequate (as performed in Equations (42) and (43). This may, however, be inadequate if the goal is to compare with the partition coefficient obtained experimentally. For a quantitative comparison with experimental data, the pre-exponential factor should be the thickness of the membrane considered in the experimental characterization of the partition coefficient. In MD simulations the membrane is reduced to one dimension, justified by the assumption that the membrane in MD simulations is flat and laterally homogeneous. Therefore, for the quantitative comparison between the partition coefficients obtained by integration of $\Delta \Delta G^{o}\left(z\right)$ and the experimental $K_{P}$, the pre-exponential factor should be the membrane thickness (h) that corresponds to the lipid molar volume considered experimentally (${\bar{V}}_{L}$). The thickness of each membrane leaflet may be calculated with Equation (44):

(44) $$h\left(nm\right)=\frac{{\bar{V}}_{L}\left({dm}^{3}{mol}^{-1}\right)}{a_{L}\left({nm}^{2}\right)N_{A}\left({mol}^{-1}\right)10^{-24}\left({{dm}^{3}nm}^{-3}\right)}$$

where $a_{L}$ is the area per lipid, and $N_{A}$ is the Avogadro constant. For membranes composed of phosphatidylcholine lipids in the fluid phase and acyl chains with 16 or 18 carbons (e.g., POPC), ${\bar{V}}_{L}$ ≅ 0.8 dm^3^ mol^−1^ [57] and $a_{L}$ = 0.64 nm^2^ at 30 °C [73]. This leads to h ≅ 2 nm for each membrane leaflet, in very good agreement with the bilayer thickness measured experimentally [73].

The integration limits and pre-exponential factors used in Equation (42) that provide consistency with the partition coefficients measured experimentally are therefore integration through the membrane plus interface until the water regions where $\Delta G^{o}$ is constant (where $\Delta \Delta G^{o}=0$) on both sides of the bilayer, and the pre-exponential should correspond to the sum of both monolayer thicknesses calculated from Equation (44) considering the molar volumes used to obtain the experimental partition coefficient, Equation (45).

(45) $$K_{P}=\frac{1}{h_{1}+h_{2}}\int_{-z_{\Delta \Delta G^{o}=0}}^{z_{\Delta \Delta G^{o}=0}}K\left(z\right)\;dz$$

In the case of symmetric membranes, this is equivalent to integrating only on one of the membrane leaflets, from the bilayer midplane to water, Equation (46).

(46) $$K_{P}=\frac{1}{h}\int_{z=0}^{z_{\Delta \Delta G^{o}=0}}K\left(z\right)\;dz$$

The partition coefficients obtained from Equation (46) for the hypothetic solutes are represented in Figure 10. As expected, the partition coefficient obtained for the small and very hydrophobic solute that may be located anywhere in the membrane nonpolar region is close to the partition coefficients obtained from the minimum energy (Equation (41))—$LogK_{P}=$ 4.5 and 4.6, respectively. However, for the solutes that are located at a well-defined depth in the membrane, the two estimates of $K_{P}$ may differ by orders of magnitude, with Equation (41) overestimating the solute–membrane affinity.

A note should be given regarding the fraction of lipid accessible to the solute (Section 2.2). While all lipids are accessible in MD simulations, limited accessibility may be observed experimentally depending on the rate of solute equilibration and if the liposomes present multilamellarity, leading to an underestimation of the solute–membrane affinity obtained experimentally.

An additional concern when comparing the solute–membrane affinities obtained experimentally and by MD simulations is related to the assumption that the simulated membrane is flat and laterally homogeneous, allowing the quantification of the relative amount of solute in the membrane simply from its probability density at a given z coordinate. In fact, significant lateral heterogeneity and local membrane curvature may be observed when performing biased simulations for very polar or strongly amphiphilic solutes [381,382,383,384,385,386]. When enhanced sampling methods are used that force solutes with polar groups to sample the nonpolar region of the membrane, the flexible membrane deforms locally, maintaining the interactions between the solute and the lipid polar groups in spite of the low average value of the *z* coordinate. In turn, when the COM of amphiphilic solutes sample *z* coordinates which on average correspond to the water medium, the membrane deforms to shield the solute nonpolar regions from the water. These effects lead to artificial free energy profiles, which may severely influence the solute–membrane affinity calculated.

**Figure 11:**
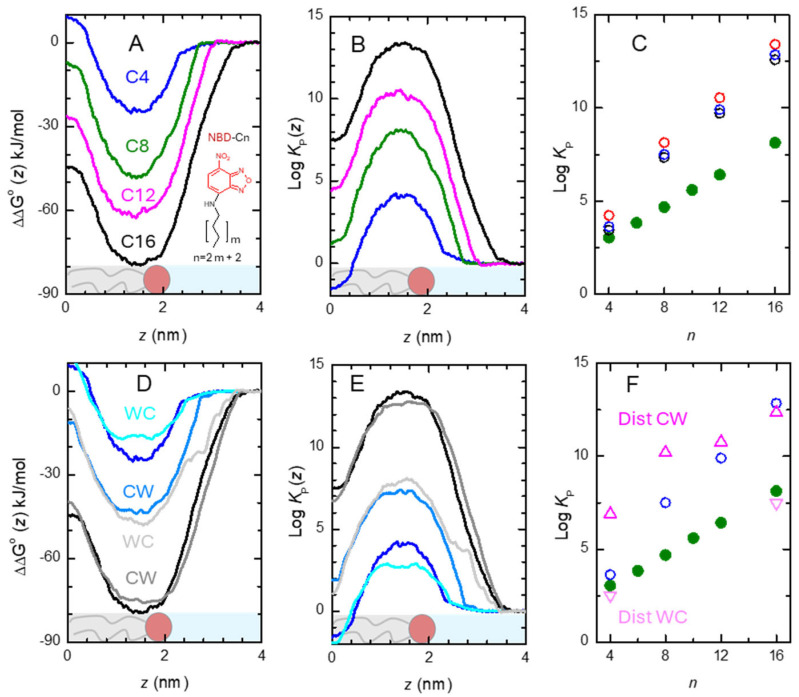
Effect of the methodology followed on biased MD simulations through umbrella sampling with the COM of the NBD polar group constrained at distinct z coordinates (data from reference) [381]. Plot (**A**): Free energy profiles obtained with the z coordinate calculated with respect to the membrane mid-plane in a cylinder centered in the solute, and with the solute pulled from the membrane mid-plane towards the water (CW) for NBD-Cn with *n* = 4 (**—**), 8 (**—**), 12 (**—**), and 16 (**—**). The corresponding local $K_{P}$ calculated from Equation (40) is shown in Plot (**B**), and the overall $K_{P}$ is shown in Plot (**C**), when calculated from the minimum in $\Delta \Delta G^{o}$, Equation (41) (◯); and from Equation (46) with integration from z = 0 to $\Delta \Delta G^{o}$ = −0.2 kJ/mol and a pre-exponential factor equal to the integration width (◯); and with the pre-exponential factor equal to the thickness of the membrane leaflet, h  = 2 nm (◯); for comparison, the experimental values of $K_{P}$ (●) are also shown. Plot (**D**): Free energy profiles obtained with the z coordinate calculated as the distance to the mid-plane membrane of the whole simulated membrane and with the solute pulled from the membrane mid-plane towards the water (CW) for NBD-Cn with *n* = 4 (**—**) and 16 (**—**), or from the water to the membrane mid-plane (WC) for NBD-Cn with *n* = 4 (**—**) and 16 (**—**); for comparison, the free energy profiles obtained from the cylinder and CW approach of the solutes are also shown: (**—**) and (**—**) for *n* = 4 and *n* = 16, respectively. The corresponding local $K_{P}$ calculated from Equation (40) is shown in Plot (**E**), and the overall $K_{P}$ calculated from Equation (46) is shown in Plot (**F**) for the CW (Δ) and WC (∇) pulling approach and compared with those obtained with the cylinder and CW approach (◯) and with the experimental values of $K_{P}$ (●).

An example is illustrated in Figure 11 for the case of a homologous series of NBD-Cn amphiphiles with a polar region (the NBD group) and alkyl chains of different length (Cn). The results from MD simulations are taken from reference [381] and compared with experimental results from reference [387]. The results shown in the upper plots were obtained with the coordinate z calculated with respect to the membrane mid-plane in a small cylinder centered in the solute, with this approach being followed to prevent membrane deformation when the solute is at the membrane/water interface. For the less hydrophobic solute (with a 4 carbon alkyl chain, NBD-C4), $K_{P}$ calculated with Equation (46) is in reasonable agreement with that obtained experimentally, while Equation (41) leads to an overestimation as expected—${LogK}_{P}=$ 3.0, 3.6, and 4.2 for experimental results and calculated from Equations (46) and (41), respectively. The agreement between the membrane affinity calculated from the free energy profile obtained in the MD simulations and that experimentally obtained progressively worsens as the amphiphiles’ hydrophobicity increases (longer alkyl chains), and for NBD-C16, even Equation (46) overestimates $K_{P}$ by more than four orders of magnitude. When the z coordinate is calculated with respect to the mid-plane of the whole membrane, severe membrane deformations are observed, and the free energy profiles are strongly dependent on the pulling method (CW—from the membrane center to water, or WC—from water to the membrane center) (lower plots). The positive curvature generated in the membrane when following the CW pulling method leads to an increase in the calculated $K_{P}$, while a membrane thinning and a negative curvature are observed in the WC pulling method that leads to a decrease in the calculated $K_{P}$.

The example shown in Figure 11 illustrates the importance of preventing membrane deformation when performing biased MD simulations to obtain free energy profiles and partition coefficients. The best approach in this respect would be to perform unrestrained MD simulations. However, adequate sampling at z coordinates with large $\Delta G^{o}\left(z\right)-\Delta G^{o}\left(z_{Eq}\right)$ may require unfeasibly long simulation times. An estimate of the time required to cross a given energy barrier for a sufficient number of times to be statistically significant (≫10 times) could be calculated from the absolute rate theory [388,389]. However, this requires knowledge of the transmission coefficient (κ) which, in condensed media, may be much smaller than unity [390,391,392,393]. An estimate of $\kappa \approx 10^{-5}$ was obtained for the case of cholesterol translocation through POPC:Chol bilayers using long unrestrained coarse-grained simulations, where an energy barrier of ≈ 10 kJ/mol results in a rate constant of 3 µs^−1^ [394]. If a similar value of κ is assumed for insertion/desorption events, only ≈ 30 events would be observed per solute molecule during a 10 µs unrestrained simulation and an energy difference of 10 kJ/mol between the solute in water and at the equilibrium position in the membrane. This approach has been followed recently to characterize the interaction of the MRI contrast agent [Gd(DOTA)]^−^ with POPC membranes [395], with ≈ 60 insertion/desorption events being observed during a total of 24 µs simulation (6 simulations of 1 µs each with 4 solute molecules), a residence lifetime of ≈ 300 ns in the membrane and 250 ns in the aqueous phase. The solute stabilization, when associated with the membrane, was ≅ 5 kJ/mol, leading to $K_{P}$ ≈ 2 when calculated from Equation (46). These results suggest that the transmission coefficient for transformations involving the dense membrane/water interface is similar or smaller than that observed for cholesterol translocation. A residence lifetime close to 1 µs is therefore expected for energy differences of 10 kJ/mol and over 100 µs for 20 kJ/mol. Direct characterization of solute partition with unrestrained simulations is therefore currently limited to solutes with low-to-moderate hydrophilicity and lipophilicity (${LogK}_{P}≅0\;\pm 1$ or 0 ±2) for simulations of several hundreds of µs, preventing its application to characterize the membrane affinity of most drugs.

Convergence of the free energy profile obtained is of particular importance in both unrestrained and biased simulations. In the first, this may be evaluated through the simulation of independent replicates and/or several solute molecules in each simulation with the solute initially located in the aqueous media or associated with the membrane (e.g., in the bilayer center). The obtention of the same final parameters for the distinct initial conditions considered is an indication of adequate convergence and gives confidence in the results obtained [214,233,395]. Conversely, distinct average parameters for each replicate and/or distinct solute molecules in each replicate point towards convergence issues and limit the validity of the average parameters obtained [3,214,396]. In biased simulations, convergence may be evaluated through the dependence of the free energy profile on the simulation time, with systematic trends pointing towards convergence issues, and small oscillations around an average profile being indicative of good convergence [190,214,381,382]. It should be noted that in the case of biased simulations, adequate convergence cannot be guaranteed simply through an extension of the simulation time. This is particularly relevant in umbrella sampling simulations where significant membrane deformation may be achieved at long simulation times when the solute is constrained at some locations in the membrane [381,397,398,399,400].

In unrestrained simulations, the probability density of the solute at each z coordinate is obtained directly from the fractional time spent by the solute at that coordinate. The local partition coefficient for a specific depth in the membrane (*z*) may then be obtained directly from the relative solute probability density ($p_{S}$) or the relative time spent at that z coordinate (Equation (47)) [372,394,395].

(47) $$K\left(refz\right)=\frac{p_{S}\left(z\right)}{p_{S}\left(z_{ref}\right)}=\frac{\Delta t\left(z\right)}{\Delta t\left(z_{ref}\right)}$$

These local partition coefficients are equivalent to those obtained with Equation (40). The excess Gibbs free energy at the z coordinate may also be calculated from the relative solute probability density (Equation (48)) and all previously discussed approaches may be used to calculate the partition coefficient from the Gibbs free energy profile.

(48) $$\Delta \Delta G^{o}\left(z\right)=-RTln\left(\frac{p_{S}\left(z\right)}{p_{S}\left(z_{ref}\right)}\right)$$

The overall partition coefficient may also be calculated directly from the probability density, in analogy with what is done experimentally (Equation (49)) [395], where $p_{S}\left(M\right)$ and $p_{S}\left(W\right)$ are the probability density of finding the solute in any coordinate within the membrane or in the water, respectively, and $\langle z_{M}\rangle$ and $\langle z_{W}\rangle$ correspond to the thickness of the membrane and water layers in the simulation box.

(49) $$K_{P}=\frac{\frac{p_{S}\left(M\right)}{\langle z_{W}\rangle }}{\frac{p_{S}\left(W\right)}{\langle z_{W}\rangle }}=\frac{p_{S}\left(M\right)\langle z_{W}\rangle }{p_{S}\left(W\right)\langle z_{M}\rangle }$$

The location of the membrane/water interface may be defined in terms of the dependence of $p_{S}\left(z\right)$ with the z coordinate, with water corresponding to the region where $p_{S}\left(z\right)$ is independent on z (that is, where the solute is not affected by the presence of the membrane). In the case of the very polar contrast agent [Gd(DOTA)]^−^, the transition occurs only at z= 4 nm, with a local maximum in $p_{S}\left(z\right)$ being observed at z≅ 2.8 nm, corresponding to the solute adsorbed at the membrane/water interface, and a global maximum in $p_{S}\left(z\right)$ observed at z≅ 1.6 nm, corresponding to the solute inserted in the membrane [395]. Both solute populations should be included in the calculation of the overall $K_{P}$ because both contribute to the amount of solute associated with the membrane obtained experimentally. However, as discussed above, the division of $p_{S}\left(M\right)$ by the width of the total region where the solute is affected by the membrane ($\langle z_{M}\rangle$) is not in agreement with that assumed experimentally. Following the same reasoning as before, the width considered for the membrane should be that calculated from Equation (44). In any case, the different values considered for $\langle z_{M}\rangle$ introduce only small variations in the calculated $K_{P}$, up to a factor of 2 in this case.

It should be mentioned that Equations (43), (46), and (49) are equivalent; they are simply expressed with regard to distinct solute and system properties [395]. In all cases, to obtain an estimate of the fraction of solute associated with the membrane, it is necessary to sum up from z = 0 until the z coordinate, where the solute is no longer influenced by the presence of the membrane ($\left|\Delta \Delta G^{o}\right|≅0$). However, if the objective is to compare with experimentally obtained partition coefficients, the solute concentration in the membrane should be calculated assuming the same membrane volume as considered experimentally. Therefore, when using Equations (43) and (49), the number of membrane windows or its corresponding width should be equal to that assumed experimentally. This is taken into consideration in Equation (46) by using the appropriate monolayer thickness h in the pre-exponential.

Independent of the analysis details discussed above, the adequacy of the force fields (FFs) used in the simulations is of utmost importance for the quality and adequacy of the free energy profiles obtained and, therefore, for the partition coefficient obtained. There are several FFs available for MD simulation of lipid bilayers and their interaction with small molecules, including several levels of detail from all-atom to coarse-grained, some of which accounts for explicit polarization. Although they are typically validated against experimental data during development (namely lipid bilayer structural and dynamical parameters), different membrane properties are described with varying accuracy by distinct FFs. A considerable amount of research has been dedicated to the comparison of FF performance in the simulation of different lipid bilayers [401,402,403,404,405,406,407,408,409,410]. While no single FF provides complete agreement with all experimental data, these studies revealed that certain bilayer properties and regions are better described by some FFs than others. For computational estimation of partition, modeling of the small molecule(s) under study is also crucial, and suitable tools are currently available for generating parameters appropriate to the major forcefields [411,412,413].

The variety of FF choices offered implies that free energy profiles and partition coefficients obtained for a given solute/lipid bilayer combination with different FFs may differ appreciably. This has been demonstrated in a benchmark study [414], employing MD with all-atom (Slipids, CHARMM36, GAFFlipids) and united-atom (GROMOS 43A1-S3, Berger) FFs, as well as the COSMOmic method (discussed in Section 3.2.2), in the calculation of the free energy profiles of 11 small molecules (glycerol, methanol, acetone, 1-butanol, benzylalcohol, aniline, 2-nitrotoluene, p-xylene, 4-chloro-3-methylphenol, 2,4,5-trichloroaniline, hexachlorobenzene) across a DMPC bilayer. While all strategies lead to significant (R^2^ > 0.7) positive correlations between calculated and experimental partition coefficients, mean absolute differences vary among different FFs by as much as a log unit. For the particular set of systems addressed in that study, Slipids was shown to be the most accurate method, followed by COSMOmic, CHARMM36, GAFFlipids, GROMOS 43A1-S3, and Berger. All-atom FFs and COSMOmic reproduced the log *K* with a mean absolute difference of <0.8 log units. Another study compared the Drude polarizable FF with the nonpolarizable CHARMM36 in the simulation of eight amino acid side chain analogs interacting with a POPC bilayer [414]. The free energy profiles obtained with the two methods are similar for nonpolar, uncharged molecules, as well as for the methylguanidinium cation. However, for more polarizable solutes, such as benzene or the acetate anion, important differences were noticed, pointing to the possible advantage in using the more computationally expensive explicit polarization FF in these situations.

For some final remarks, to obtain a quantitative agreement between the partition coefficient estimated from in silico approaches and those obtained in vitro or in vivo, several fundamental aspects must be addressed. At first, the choice of the most appropriate force fields, which may require the inclusion of polarization; the need to guarantee system equilibration regarding solute orientation and conformation when associated with the membrane; convergence of the free energy profiles when using biased MD simulations [6,15,379,415,416,417,418]; and, for a full agreement, other aspects such as contributions from the distinct translational entropy in the two media [419,420,421,422,423] may also need to be included. Another important aspect usually overlooked is the need to consider possible changes in the ionization of the solute in the highly heterogeneous membrane [424,425,426,427,428,429]. Given its relevance, this aspect is specifically addressed in Section 3.2.3.

#### 3.2.2. Implicit Solvation Models

Despite their obvious utility, molecular dynamics simulations with explicitly defined lipid and solvent molecules involve considerable computational cost, namely when calculation of the free energy for the interaction of solutes with the membrane is required, as is the case for partition. For this reason, methods that employ a simplified implicit representation of water and/or lipid molecules have been proposed. Two such approaches are described here, based on the generalized Born (GB) and conductor-like screening (COSMO) solvation models.

Using a mean-field approach to represent the environment, the electrostatic component of solvent–solute interactions can be calculated by numerically solving the Poisson–Boltzmann equation, or slightly less accurately but more efficiently, using analytical approximations such as the GB method [430]. According to this formalism, the electrostatic contribution $\Delta G_{elec}^{o}$ to the solvation free energy $\Delta G_{solv}^{o}$ of a given solute is described by a sum of pairwise terms of interaction between atoms i and j, separated by a distance $r_{ij}$, with charges $q_{i}$ and $q_{j}$, respectively [431].

(50) $$\Delta G_{elec}^{o}=-\frac{1}{2}\left(\frac{1}{\epsilon_{r}^{1}}-\frac{1}{\epsilon_{r}^{2}}\right)\sum_{i,j}\frac{q_{i}q_{j}}{f_{ij}^{GB}}$$

In Equation (50), $\epsilon_{r}^{1}$ and $\epsilon_{r}^{2}$ are the relative solute and solvent dielectric constants, respectively, the absolute permittivity and additional pre-factors [432] being absorbed into the Coulomb constant used by the force field, and

(51) $$f_{ij}^{GB}(r_{ij})=\sqrt{r_{ij}^{2}+R_{i}R_{j}exp\left\{-r_{ij}^{2}/(4R_{i}R_{j})\right\}}$$

In the latter equation, $R_{i}$ and $R_{j}$ are distance parameters denoted effective Born radii for atoms i and j, respectively.

Tanizaki and Feig [433] adapted this formalism to a “heterogeneous dielectric generalized Born” (HDGB) model of a lipid bilayer. In brief, this extension was made by considering that ϵ in the bilayer phase depends on the transverse location z, and considering an apparent local dielectric constant profile, $\epsilon_{r}^{\prime }\left(z\right)$). Thus, the electrostatic interaction for a solute atom pair ij is characterized by a dielectric constant given by the following:

(52) $$\epsilon_{ij}=\frac{\epsilon_{r}^{\prime }\left(z_{i}\right)+\epsilon_{r}^{\prime }(z_{j})}{2}$$

The $\epsilon_{r}^{\prime }\left(z\right)$ function is computed numerically by solving the Poisson equation as a probe ion moves across an implicit membrane/water model, most efficiently made up of three regions with different dielectric constant values (three-dielectric model; e.g., $\epsilon_{r}=2$ in the bilayer core, 7 near the lipid/water interface, and 80 in bulk water).

The electrostatic component is then added to the nonpolar contribution to the solvation free energy ${\Delta G}_{np}^{o}$, the latter made up by summing the van der Waals solute–solvent interactions (${\Delta G}_{vdw}^{o}$) and the cost of cavity formation (${\Delta G}_{cavity}^{o}$), both assumed to depend linearly on the solvent-accessible area (${SA}_{i}$ for atom i):

(53) $${\Delta G}_{np}^{o}={\Delta G}_{vdw}^{o}+{\Delta G}_{cavity}^{o}=\gamma \sum_{i}S\left(z_{i}\right)SA_{i}$$

In the latter equation, γ is an empirical surface tension parameter and $S\left(z\right)$ is a profile function that accounts for the variation of the surface tension along z. In the original paper [433], this was obtained by fitting an analytical differentiable function to an MD-derived free energy profile of O_2_ insertion, assuming that SA is independent of z for this solute. The HDGB model was then applied to the calculation of the free energy profile of water and amino acid side chain analogs (averaging ${\Delta G}_{solv}^{o}$ for different orientations at a given fixed center-of-mass z) and also incorporated in Langevin MD simulations of melittin and bacteriorhodopsin.

More recently, an extension of the HDGB was proposed to account for the possibility of membrane deformation upon insertion of charged solutes [434]. This introduced an additional term ${\Delta G}_{mem}^{o}$ to be added to ${\Delta G}_{elec}^{o}$ and ${\Delta G}_{np}^{o}$. The term ${\Delta G}_{mem}^{o}$ can be computed by integrating over the membrane plane the contributions from compression, bending and surface tension, characterized by moduli $K_{a}$, $K_{c}$, and α, respectively [435,436]:

(54) $$\Delta G_{mem}^{o}=\iint \frac{1}{2}\left\{\frac{K_{a}}{d_{0}}u^{2}+\frac{1}{2}K_{c}{\left(\nabla^{2}u\right)}^{2}+\frac{1}{2}\alpha K_{c}{\left(\nabla u\right)}^{2}\right\}d\Omega$$

Here, u is the deviation of a membrane leaflet from its unperturbed width ($d_{0}/2$). Solutes are considered cylindrical inclusions, with the assumption of independent (and therefore additive) deformation energies in the two membrane leaflets. u is obtained by minimization of the deformation energy, leading to a differential equation that is solved with the leaflet deformation and the contact slope around the contact curve as boundary conditions, together with the assumption that the membrane is flat far from the solute. The extended formalism, termed dynamic HDGB (DHDGB), performs similarly to HDGB for noncharged amino acids but improves agreement with MD free energy profiles of the insertion of the charged ones, avoiding the overestimation present in the previous model. Both HDGB and DHDGB were later applied to the calculation of free energy profiles for insertion of a variety of drug solutes, which were conjugated with diffusivity estimates for calculation of permeability coefficients [437].

COSMOmic is an alternative emerging approach for the computation of free energy profiles for solute interaction with anisotropic systems such as micelles and lipid bilayers using a continuum dielectric solvent approach. It was originally introduced by Klamt et al. [438] as an extension of the conductor-like screening model for realistic solvation (COSMO-RS) quantum chemical method. COSMO-RS uses the COSMO solvation formalism to model electrostatic solvent–solute interactions [439], combined with equilibrium statistical thermodynamics, to calculate chemical potentials in solution. While COSMO-RS is useful to estimate partition between immiscible liquids (e.g., [440]), it does not account for the transverse anisotropy of membranes. COSMOmic achieves this by considering the dependence of the chemical potential $\mu^{X}$ of solute X on its transverse position $r^{X}$ and orientation $d^{X}$ [438]:

(55) $$\mu^{X}\left(r^{X},d^{X}\right)=\mu_{CRS}^{X}\left(r^{X},d^{X}\right)+\mu_{comb}^{X}\left(r^{X},d^{X}\right)+\mu_{elast}^{X}\left(r^{X},d^{X}\right)+\mu_{\zeta }^{X}\left(r^{X},d^{X}\right)$$

In Equation (55), the CRS term refers to the location- and orientation-dependent chemical potential calculated from the screening of charge density, which is dominant for polar solutes. The use of COSMO-RS in this calculation requires information on the distribution of the atom types over the bilayer normal, as well as the structure of a representative solute and lipid molecule. In the original COSMOmic article, lipid atom distributions and the structure of a single lipid conformer were retrieved from a sole MD simulation frame of the hydrated bilayer (note that no solute is required, since the structure of the latter is usually obtained from quantum chemical methods such as DFT). More recently, an improved implementation, using lipid atom distributions resulting from the whole MD trajectory and selection of an individual lipid molecule with a solvent accessible surface (SAS) equal to the ensemble average value of this property has been proposed [49].

The second term in the right-hand side, also present in ordinary COSMO-RS, is the so-called combinatorial contribution, which takes into account size and shape ratios of solute and solvent molecules. The third and fourth terms were purposely introduced to account for the elastic deformation and membrane electrostatic zeta potential contributions to the chemical potential, respectively. In the original COSMOmic paper [438], the elastic term was shown to not lead to significant improvement and therefore has been neglected in subsequent reports. On the other hand, the zeta potential term was not included in the calculations described in the original COSMOmic paper (which thus ended up being used only for neutral solutes), because of unrealistically strong zeta potentials estimated from MD simulations [438]. This issue was solved in subsequent reports with ionic solutes through the implementation of an empirical model potential with a small number of adjustable parameters [441].

Inclusion of bilayer transverse anisotropy is achieved by modeling the bilayer (or micelle) as a stratified liquid, with n layers of typical width 0.1 to 0.2 nm. For each layer, a large number m of possible solute orientations is considered. The probability $p^{X}$ of finding the solute in layer i (of central position $z_{i}$) is given by the following:

(56) $$p^{X}\left(z_{i}\right)=\frac{\sum_{j=1}^{m}exp\left(-\beta \mu^{X}\left(z_{i},d_{j}\right)\right)}{\sum_{k=1}^{n}\sum_{j=1}^{m}exp\left(-\beta \mu^{X}\left(z_{k},d_{j}\right)\right)}$$

where $\beta ={\left(RT\right)}^{-1}$. It follows that the free energy profile, $\Delta G^{X}\left(z_{i}\right)$, is calculated as follows:

(57) $$\Delta G^{X}\left(z_{i}\right)=-RT\;ln\frac{p^{X}\left(z_{i}\right)}{p^{X}\left(z_{n}\right)}$$

In Equation (57), the position $z_{n}$ is that of the outermost layer, corresponding to bulk water. From the free energy profile, the partition coefficient is then calculated using Equation (43).

Generally, good correlations have been obtained between experimental and COSMOmic estimates for membrane/water partition coefficients [49,438,441,442,443,444]. It should be mentioned that quantitative absolute agreement requires subtraction of a constant fitted offset value (ca. 0.32 log units in reference [441]). Additionally, the partition coefficients of more hydrophilic solutes are commonly underpredicted [444,445].

Still, from the computational point of view, COSMOmic provides a highly efficient way to predict partition coefficients between water and anisotropic media such as membranes. The partition behavior of each solute (notably including the free energy profile) takes a few seconds, provided that all necessary inputs are in place. The latter typically require MD simulations for the atom distributions and lipid conformer structure (experimental data could be used in principle, though so far this has not been the case) and quantum chemical approaches such as DFT calculations for both the lipid and solute. In any case, taking into account that a single MD simulation of a specific bilayer is necessary to predict the partition of any solute in this system, this makes for a considerable gain in speed compared to enhanced-sampling MD. Thus, COSMOmic calculations constitute a very attractive approach for rapid computational estimation of free energy profiles and partition coefficients.

#### 3.2.3. Inclusion of Changes in Solute Ionization upon Membrane-Association

The computational methods described in Section 3.2.1 and Section 3.2.2 do not take into consideration possible changes in the solute ionization when partitioning from the aqueous media to the lipid membrane. This is a major limitation given the common deviation observed in the solute pKa upon membrane association (see Quantification of the solute from changes in its ionization equilibria section). To overcome this limitation, the free energy profile of the most relevant ionization forms of the solute may be obtained using independent simulations. The partition coefficient of each species may then be obtained from the respective free energy profile, following the formalisms discussed in Section 3.2.1. If the ionization constants in the aqueous media ($K_{a}^{W})\;$are known, these simulations provide all the necessary parameters to describe the interaction of the solute with the lipid bilayer at a given pH value. In the case of a single ionizable group; the full characterization of solute interaction with the membrane involves four species (the protonated and unprotonated species in the water and associated with the membrane, ${SH}_{W}$, $S_{W}$, ${SH}_{M}$, and $S_{M}$, respectively) and four equilibrium constants ($K_{P}^{SH}$, $K_{P}^{S}$, $K_{a}^{W}$, and $K_{a}^{M}$) (Figure 6). The simulations provide estimates for $K_{P}^{SH}$and $K_{P}^{S}$, and if $K_{a}^{W}$ is known, the thermodynamic cycle allows calculation of $K_{a}^{M}$ (Equation (26)).

The free energy profiles of the distinct species may also allow obtaining the ionization equilibrium constant at distinct depths in the membrane ($K_{a}^{z}$). For this goal, it is first necessary to shift the energy profiles of the distinct ionization forms to match their free energy difference in the aqueous media predicted from $K_{a}^{W}$. This procedure also allows the calculation of the ionization constant at the equilibrium position in the membrane ($K_{a}^{z_{Eq}}=K_{a}^{M})$. The overall free energy profile at a given pH in the aqueous media may also be calculated, corresponding to the minimum free energy path, from which the overall partition coefficient may then be calculated and compared with that obtained experimentally at a given pH value. The overall free energy profile can also be used to calculate the rate of solute permeation through the lipid membrane, but this is beyond the scope of this revision (interested readers may refer to the recent reviews [427,446]).

The approach described above has been followed to obtain the relative stability of the neutral and ionized species of several drugs at different depths in the membrane and used to calculate the permeability coefficient at different pH values [17,428,447]. The free energy profiles of the distinct ionization forms have also been obtained for some additional solutes, including drugs [3,6,8,448,449], other bioactive molecules [17,396], and fluorescent probes [214,233,450], but the distinct free energy profiles were mostly used to obtain the relative affinity of the distinct forms for the membrane and their relative permeability coefficients.

An alternative approach to incorporating solute ionization when characterizing its interaction with membranes is following the constant pH methodology, where changes in the solute ionization are allowed during the MD simulations [428,451,452,453]. Constant-pH molecular dynamics (CpHMD) refers to a group of methods that simultaneously determine the charge states of ionizable groups during conformational dynamics. While conventional molecular dynamics simulations generally assume fixed protonation states, a constant-pH methodology addresses the dynamic shifts in protonation states during the simulation. The CpHMD methods are categorized as discrete or continuous based on how charge states are represented and sampled during the simulations. In discrete CpHMD [451], a standard MD simulation is periodically interrupted to add a step of charge state evaluation of each ionizable group using Monte Carlo methods, which determines if the randomly generated new charge states should be accepted or rejected. Then, MD simulation will continue with the new charge states if accepted and/or old charge states if rejected. In continuous CpHMD [454,455,456,457], an extended Hamiltonian of the system is used where the added degree of freedom is based on the λ-dynamics approach for free energy calculations, where each ionizable site is assigned to a λ value between 0 (protonated form) and 1 (deprotonated form) that propagates simultaneously with spatial coordinates. This formalism avoids the large energy jumps of discrete methods by allowing a progressive rearrangement of the local environment [458].

The CpHMD methods have been applied to the mechanistic understanding of a variety of biomolecular systems. Examples include the characterization of the protonation equilibria of titratable membrane lipids, solutes at the membrane/water interface [426,459,460,461,462,463] and during membrane insertion and permeation [427,428,464].

Yue et al. characterized propranolol ionization equilibria and membrane permeation using the CpHMD methods and those involving the combination of distinct fixed-ionization MD simulations, with a good agreement being obtained for the lowest free energy path from both methods [428]. In that work protonation and conformation equilibria were dynamically coupled to study the permeation of the ionizable drug through a POPC lipid bilayer. It was found that propranolol migrates into the bilayer in the charged form and deprotonates at the hydrophobic boundary and that by involving dynamic protonation, the permeation coefficient was predicted with high accuracy. This work shows that the correct determination of the location-dependent ionization is required for the correct determination of membrane partition and membrane permeation.

The use of constant-pH MD simulations is still limited despite the developments for the application in different simulation software [465,466] and force fields [429,466,467]. This is mainly due to their higher level of system preparation and computational complexity [468,469]. Fortunately, nowadays with tools such as the pHbuilder for the automatic preparation of constant-pH MD simulations in GROMACS [470], it is expected that constant pH simulations become easier to set up and run, thereby making them more accessible to the scientific community.

## 4. Conclusions

There is a large and varied ensemble of methodologies to quantify the association affinity of small molecules, such as drugs, for model lipid membranes. In this work, the advantages and limitations of experimental and computational approaches have been systematically analyzed, and the diversity of affinity parameters reported in the literature has been rationalized. Particular attention has been given to the assumptions, formalisms, and experimental conditions underlying each methodology, as these factors ultimately determine the validity and comparability of the obtained results. By explicitly addressing these aspects, this review aims to clarify the origins of apparent discrepancies between methods and to define the conditions under which meaningful consensus parameters may be established.

Beyond a critical comparison of methodologies, this integrative perspective contributes to the establishment of robust biochemical and biophysical foundations for solute–membrane interactions. Such foundations are essential for relating experimentally accessible affinity parameters to the molecular properties of small molecules, including size, polarity, charge, and chemical functionality. In this context, the combined use of carefully designed experiments and appropriately parameterized computational models emerges as a promising strategy to bridge molecular-scale descriptions and macroscopic observables. Ultimately, the framework presented here provides a basis for the development of quantitative and predictive relationships aimed at forecasting membrane affinity from molecular structure, thereby supporting both fundamental studies and applied efforts in drug design and membrane biophysics.

## Data Availability

The original contributions presented in this study are included in the article. Further inquiries can be directed to the corresponding author.

## Abbreviations

The following abbreviations are used in this manuscript:

ADIFAB	Acrylodan labeled intestinal fatty acid binding protein)
ADME/Tox	Absorption, distribution, metabolism, elimination and toxicity
CW	Pulling from the membrane center to the aqueous medium
COM	Center of mass
COSMO	Conductor-like screening
COSMO-RS	Conductor-like screening model for realistic solvation
COSMOmic	Conductor-like screening model for realistic solvation for micelles
CpHMD	Constant-pH molecular dynamics
DFT	Density functional theory
DHDGB	Dynamic heterogeneous dielectric generalized Born
di-8-ANEPPS	3-[4-[(E)-2-[6-(dioctylamino)naphthalen-2-yl]ethenyl]pyridin-1-ium-1-yl]propane-1-sulfonate
DLS	Dynamic light scattering
DOPC	1,2-dioleoyl-*sn*-glycero-3-phosphocholine
DOTA	2,2′,2″,2‴-(1,4,7,10-Tetraazacyclododecane-1,4,7,10-tetrayl)tetraacetate
DPPC	1,2-dipalmitoyl-*sn*-glycero-3-phosphocholine
EPR	Electron paramagnetic resonance
FCS	Fluorescence correlation spectroscopy
FRET	Förster resonance energy transfer
GB	Generalized Born
HDGB	Heterogeneous dielectric generalized Born
ITC	Isothermal titration calorimetry
LUV	Large unilamellar vesicle
MD	Molecular dynamics
MLV	Multilamellar vesicle
MRI	Magnetic resonance imaging
NBD	7-nitrobenz-2-oxa-1,3-diazol-4-yl
NMR	Nuclear magnetic resonance
NPT	Isothermal–isobaric ensemble
POPC	1-palmitoyl-2-oleoyl-sn-glycero-3-phosphocholine
QCM	Quartz crystal microbalance
SPR	Surface plasmon resonance
UV-vis	Ultraviolet-visible
WC	Pulling from the aqueous medium to the membrane center
